# The Use of TF-Ag-α to Selectively Capture Tumor-Derived Extracellular Vesicles and Enhance the Predictive Performance of Extracellular Vesicle PD-L1 for Response to Immune Checkpoint Inhibitors

**DOI:** 10.1158/2767-9764.CRC-26-0214

**Published:** 2026-11-02

**Authors:** Chang-Chieh Hsu, Man Qi, Fumito Ito, Grace K. Dy, Kate Rittenhouse-Olson, Yun Wu

**Affiliations:** 1 Department of Biomedical Engineering, University at Buffalo, The State University of New York, Buffalo, New York.; 2 Department of Surgery, Keck School of Medicine, University of Southern California, Los Angeles, California.; 3 Department of Medicine, Roswell Park Comprehensive Cancer Center, Buffalo, New York.; 4 For-Robin, Inc., Buffalo, New York.

## Abstract

Only a subset of patients with non–small cell lung cancer (NSCLC) benefits from immune checkpoint inhibitors (ICIs). Tissue PD-L1 has limited accuracy in predicting ICI response and requires invasive biopsies, highlighting the urgent need for reliable, minimally invasive biomarkers. Extracellular vesicle (EV) PD-L1 is a promising liquid biopsy marker, but its predictive performance is confounded by PD-L1 derived from nontumor cells. We developed a tumor-specific EV-based assay to predict ICI response. Tumor-derived EVs (TEX) were selectively captured using the tumor-specific Thomsen–Friedenreich glycoantigen α (TF-Ag-α), and PD-L1 levels in TEX expressing TF-Ag-α (i.e., TEX^TF-Ag-α^ PD-L1) were measured in sera from 91 patients with NSCLC receiving anti–PD-1 therapy (40 responders and 51 nonresponders). Predictive performance was evaluated at pretreatment baseline, at the end of the first treatment cycle, and via fold changes (FC). At pretreatment baseline, TEX^TF-Ag-α^ PD-L1 showed moderate predictive ability [area under the curve (AUC): 0.76; sensitivity: 0.97; specificity: 0.56]. By the end of the first treatment cycle, responders exhibited a ∼4.18-fold increase in TEX^TF-Ag-α^ PD-L1 expression, whereas nonresponders showed an approximate 20% decrease. Dynamic FCs after the first treatment cycle substantially improved predictive performance, achieving an AUC of 0.93, sensitivity of 0.95, and specificity of 0.80 for distinguishing responders from nonresponders, outperforming both total EV PD-L1 and tissue PD-L1. These results demonstrated that selective TEX capture using TF-Ag-α significantly enhanced the predictive value of EV PD-L1. Dynamic changes in TEX^TF-Ag-α^ PD-L1 provided a highly accurate, minimally invasive biomarker for early assessment of anti–PD-1 response, supporting tumor-specific EV-based liquid biopsy for precision immunotherapy.

**Significance:**

Reliable biomarkers for predicting ICI response remain lacking. We developed a tumor-specific liquid biopsy assay that selectively captures TEX via TF-Ag-α, enabling accurate PD-L1 measurement. By reducing interference from nontumor EVs, the assay achieved 93% predictive accuracy, outperforming tissue PD-L1 and supporting precision immunotherapy.

## Introduction

Lung cancer causes about 25% of all cancer deaths worldwide. Although immune checkpoint inhibitors (ICIs), such as pembrolizumab and nivolumab, have revolutionized treatment for various cancers, including non–small cell lung cancer (NSCLC), only a subset of patients receives durable clinical benefit (1, 2). In the absence of predictive markers of response to ICI therapy, treatment choices are empirical and further complicated by unusual response patterns and potentially severe immune-related adverse effects (irAEs; refs. 3–5). Currently, tissue biopsy and imaging modalities are used to predict and monitor the response to ICI therapy. Unfortunately, PD-L1 expression in pretreatment tumor samples has a disappointing 28.9% accuracy in predicting response to ICI therapy (6, 7). Repeated tumor biopsies may show increased T-cell clonality and expression of immune cytolytic markers in responding tumors (8) and would be ideal to evaluate evolution of tumor and microenvironment during ICI therapy (9–11). However, this approach is invasive and challenging for visceral tumors such as NSCLC. Imaging is noninvasive but limited by issues such as pseudoprogression and delayed response (12, 13). New tests are needed to address these limitations, effectively predict and monitor response to ICI therapy, and improve patient care and clinical outcomes.

Liquid biopsy enables the detection of tumor-derived biomarkers in body fluids such as blood and urine, making it possible to repeatedly assess disease status over time (14). By complementing medical imaging and clinical risk factor information, it can provide a broader view of tumor biology and progression, offering a noninvasive or minimally invasive approach to help address key challenges in ICI therapy. Tumor-derived extracellular vesicles (TEX) play important roles in every step of cancer development, immune regulation, metastasis, and drug resistance. Therefore, TEX are promising biomarkers for cancer liquid biopsy in various applications spanning from early cancer detection to treatment response prediction (15).

Recent studies have shown that extracellular vesicle (EV) PD-L1 is closely correlated with responses to anti–PD-1/PD-L1 therapy (16–23). In patients with melanoma, increased levels of exosomal PD-L1 at an early stage of treatment were observed in responders to anti–PD-1 therapy (16). A recent meta-analysis study involving 1,091 patients reported that increased exosomal PD-L1 levels in the early stages of immunotherapy was associated with improved patient prognosis (23). At a later stage, such as 3 to 4 months after treatment, exosomal PD-L1 levels may decrease as tumor burden is reduced (21). However, PD-L1 is expressed not only by cancer cells but also by immune cells, including T cells, B cells, NK cells, dendritic cells, and macrophages, all of which can release PD-L1–positive EVs. EV PD-L1 derived from nontumor cells may therefore confound the detection of TEX PD-L1, reducing the predictive accuracy of PD-L1 as a biomarker for response to ICI therapy.

We recently discovered a tumor-specific EV biomarker, Thomsen–Friedenreich glycoantigen α (TF-Ag-α). TF-Ag-α is a tumor-specific form of the disaccharide TF-Ag [Gal-β-(1–3)-GalNAc], which is usually concealed in normal tissues but exposed in tumors because of altered glycosylation (24, 25). We developed a TF-Ag-α–specific monoclonal antibody, JAA-F11, and demonstrated TF-Ag-α expression across a broad range of cancers, including lung, breast, colon, bladder, ovarian, prostate, and stomach cancers (26). We further confirmed that TF-Ag-α is carried by EVs by visualizing the EVs using scanning electron microscopy and observing significantly higher TF-Ag-α levels in serum and serum-derived EVs, but not in EV-depleted serum samples (27). We showed that serum EV TF-Ag-α effectively discriminated five cancer types (lung, breast, colon, ovarian, and prostate cancers; *n* = 335) from noncancer controls (*n* = 187), achieving an overall accuracy of 96.2% (27, 28).

In this study, TF-Ag-α was used to selectively capture TEX from the total EVs. The levels of TEX TF-Ag-α and TEX^TF-Ag-α^ PD-L1 in serum samples were then measured simultaneously using our Exo-PROS biosensor (29). We enrolled 91 patients with NSCLC receiving anti–PD-1 therapy, including 40 responders [complete response (CR) or partial response (PR)] and 51 nonresponders [stable disease (SD) or progressive disease (PD)], with serum collected at pretreatment baseline and at the end of the first treatment cycle. The dynamic fold change (FC) in TEX^TF-Ag-α^ PD-L1 distinguished responders from nonresponders with a sensitivity of 0.95, specificity of 0.80, and an area under the curve (AUC) of 0.93 (cutoff = 1.59). Notably, TEX^TF-Ag-α^ PD-L1 outperformed total EV PD-L1 and tissue PD-L1 in predicting response to anti–PD-1 therapy.

## Materials and Methods

### Materials

Methyl-[polyethylene glycol (PEG)]4-thiol [PEG200-SH, molecular weight (MW): 224 Da, 26132] was purchased from Sigma-Aldrich. Biotin-PEG1000-SH (MW: 1000 Da, PG2-BNTH-1k) was purchased from Nanocs. NeutrAvidin protein (31000), biotinylated anti–PD-L1 antibody (clone MIH1, 13-5983-82, RRID: AB_466840), fluorescent dye allophycocyanin (APC)-labeled anti–PD-L1 antibody (clone MIH1, 17-5983-42, RRID: AB_10597586), and EZ-Link Micro Sulfo-NHS-LC-Biotinylation Kit (21935) were purchased from Thermo Fisher Scientific. Biotinylated anti-LG3BP antibody (LS-C686310-1) was purchased from LSBio. JAA-F11 anti–TF-Ag-α mouse antibody was obtained from For-Robin, Inc. A SYLGARD 184 silicone elastomer kit [polydimethylsiloxane (PDMS), 2065622] was purchased from Dow Corning.

### Fabrication of Exo-PROS biochip

A glass slide (12-550-A3, Thermo Fisher Scientific) underwent cleaning using acetone, 200-proof ethanol, and deionized water with 10 minutes of sonication. Using the Kurt J. Lesker AXXIS Electron-Beam Evaporator, a 2-nm layer of Ti followed by a 49-nm layer of Au was deposited onto the surface of the glass slide. The deposition rate was set at 0.8 Å/second for Ti and 1 Å/second for Au. Finally, a PDMS piece featuring a 6-mm-diameter well and an Au-coated glass slide were subjected to oxygen plasma treatment and then securely bonded together for subsequent use.

### Surface modification of Exo-PROS biochip

The surface of the Exo-PROS biochip was first modified with PEG by incubating it with a mixture of PEG200-SH and biotin-PEG1000-SH (100 μL, 10 mmol/L in PBS; PEG200-SH:biotin-PEG1000-SH molar ratio of 3:1) for 1 hour at room temperature. Excess PEG was removed by washing 3 times with PBS. NeutrAvidin (100 μL, 50 μg/mL) was then added and incubated at room temperature for 1 hour. After removal of excess NeutrAvidin by 3 PBS washes, biotinylated antibodies against TF-Ag-α, PD-L1, or LG3BP (50 μL, 50 μg/mL) were added and incubated on the biochip overnight at 4°C to immobilize the antibodies on the surface via biotin–avidin interactions. Excess antibodies were removed by washing 3 times with PBS, and the biochip was then ready for use.

### Detection of TEX TF-Ag-α and TEX^TF-Ag-α^ PD-L1 using Exo-PROS assay

We have developed an Exo-PROS assay that enables the direct capture of TEX from serum samples and the simultaneous detection of multiple TEX biomarkers on a single platform (27, 29). The Exo-PROS assay employs surface plasmon resonance (SPR) as the sensing mechanism, allowing sensitive detection of different types of TEX biomarkers including proteins, microRNAs, and carbohydrate antigens. In this study, the Exo-PROS assay was used to first quantify the levels of TEX TF-Ag-α and subsequently measure PD-L1 levels on these TF-Ag-α–expressing TEX (i.e., TEX^TF-Ag-α^ PD-L1).

**Figure 1.**
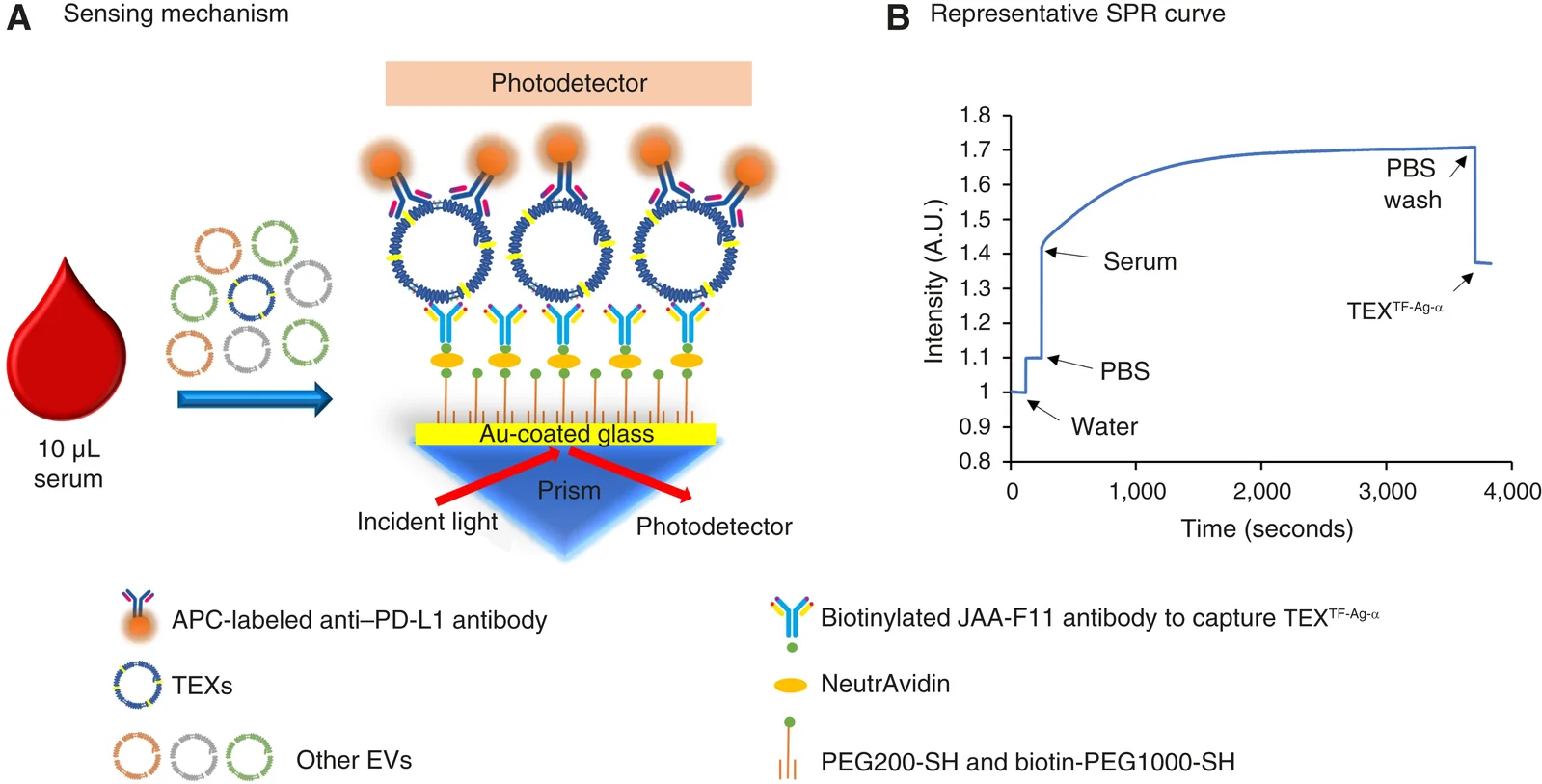
Detection of TEX^TF-Ag-α^ PD-L1 using the Exo-PROS assay. **A,** Sensing mechanism of the Exo-PROS assay. **B,** Representative SPR curve for detecting TEX TF-Ag-α in the serum sample of a patient with stage IV NSCLC. A.U., arbitrary unit.

As shown in Fig. 1, the biochip was mounted onto the Exo-PROS biosensor. A 647-nm laser with a power density of 30 mW was directed through the sample to generate SPR signals. The SPR signals of water (I_water_) and PBS (I_PBS_) were recorded for 2 minutes to establish the baseline and minimize the chip–chip variation. Human serum samples (10 μL serum diluted with 40 μL PBS) were then applied to the biochip coated with JAA-F11 anti–TF-Ag-α antibodies, in which TEX^TF-Ag-α^ levels were captured by the immobilized antibodies without the need for EV isolation step. The SPR signal acquisition was performed at room temperature for 1 hour. Nonspecific binding of EVs was removed by washing 3 times with PBS. The SPR signal from TF-Ag-α–positive EVs (I_TEX TF-Ag-α_) was subsequently recorded for 2 minutes. The expression level of TEX TF-Ag-α was calculated using Eq. A:

$$\text{TEX }\text{TF}‐\text{Ag}‐\alpha \;e\text{xpression}\;=\;\left(I_{\text{TEX TF}‐\text{Ag}‐\alpha }\;–\;I_{\text{PBS}}\right)/\left(I_{\text{PBS}}\;–\;I_{\text{water}}\right)$$ (A)

Next, to measure the level of TEX^TF-Ag-α^ PD-L1, an avalanche photodiode was positioned above the biochip, and the baseline fluorescence signal was recorded (F_before APC-PD-L1_). Fluorescent dye APC-conjugated anti–PD-L1 antibodies (200 μg/mL, 5 μL) were then added and incubated on the biochip for 1.5 hours at room temperature. After washing off the unbound APC-conjugated anti–PD-L1 antibodies, the fluorescence signal from TEX^TF-Ag-α^ PD-L1 was recorded (F_after APC-PD-L1_). In the Exo-PROS biosensor, the fluorescence signals are enhanced ∼100 times by the compact SPR system, i.e., surface plasmon–enhanced fluorescence spectroscopy. The expression level of TEX^TF-Ag-α^ PD-L1 was calculated using Eq. B:

TEXTF‐Ag‐α  PD‐L1  expression = Fafter APC‐PD‐L1 – Fbefore APC‐PD‐L1/Fbefore APC‐PD‐L1 (B)

The same procedure was used to detect TEX LG3BP and TEX^LG3BP^ PD-L1 levels using a biochip modified with anti-LG3BP antibodies.

### Detection of total EV PD-L1 using Exo-PROS assay

Similar to the detection of TEX TF-Ag-α, total EV PD-L1 was measured using a biochip modified with anti–PD-L1 antibodies. The expression level of total EV PD-L1 was calculated using Eq. C:

Total  EV  PD‐L1  expression = IEV PD‐L1 – IPBS/IPBS – Iwater (C)

### Human serum samples

Deidentified serum samples and clinical data of patients with NSCLC who received anti–PD-1 immunotherapy were obtained from the Data Bank and BioRepository Shared Resource at Roswell Park Comprehensive Cancer Center. The serum samples were transferred to the University at Buffalo on dry ice, where they were stored at −80°C. All serum samples were centrifuged at 10,000 × *g* at 4°C for 30 minutes to remove debris and then diluted with PBS at a volume ratio of 1:4 before use. Written informed consent was obtained from all participants, and the studies were conducted in accordance with recognized ethical standards and guidelines. Approvals from institutional review boards of Roswell Park Comprehensive Cancer Center and the University at Buffalo were obtained for the use of human serum samples in this study.

### Determination of response to ICI treatment

RECIST-defined objective response was used to categorize patients into responders and nonresponders. Responders were defined as patients achieving CR (disappearance of all target lesions) or PR (a ≥30% decrease in the sum of diameters), whereas nonresponders include patients with SD (neither PR nor PD) or PD [a ≥20% increase in the sum of diameters (minimum 5-mm absolute increase) or appearance of new lesions].

All patients received standard-of-care treatment and were followed throughout the full course of therapy or until death. Baseline CT scans were obtained prior to treatment initiation, followed by serial imaging to monitor therapeutic responses. Specifically, an initial response evaluation was performed via CT at the end of treatment cycle 2 or 3, with subsequent scans conducted every 2 to 3 months. The RECIST response was defined as the best overall response recorded across the entire treatment course, rather than the response observed at the first posttreatment imaging point.

### Statistical data analysis

Binary logistic regression and receiver operating characteristic (ROC) analyses were performed to compare responders and nonresponders using individual biomarkers or their combinations in SPSS (version 26, RRID: SCR_002865). For the PD-L1 tumor proportion score (TPS)–based ROC analysis, tissue PD-L1 TPS was dichotomized by classifying samples with TPS ≥ 50% as responders and TPS < 50% as nonresponders, and this binary outcome was used for subsequent ROC evaluation. Cutoffs maximizing the sum of sensitivity and specificity were first identified, and the corresponding AUC, sensitivity, and specificity were then calculated at the selected cutoff. Data from the discovery and internal validation cohorts were analyzed independently. The AUC, sensitivity, specificity, and cutoff value were calculated and reported separately for each cohort.

In the proportional stacked bar chart, patients were stratified into three predefined percentage categories (<1%, 1%–49%, and ≥50%). For each cohort (responders and nonresponders), the number of patients falling into each category was tabulated and entered into GraphPad Prism (version 8.0, RRID: SCR_002798) as a contingency table (3 rows × 2 columns). The distribution of categories within each cohort was visualized using a 100% stacked bar chart, in which each bar was normalized to the cohort total and displayed as the percentage of patients per category. Differences in the categorical distributions between responders and nonresponders were assessed using a contingency table test (*χ*^2^ test; a Fisher exact test was used when expected cell counts were small, as appropriate).

Progression-free survival (PFS) and overall survival (OS) analyses were performed using GraphPad Prism. Patients were stratified into different groups based on the TEX^TF-Ag-α^ PD-L1 FC, and the corresponding survival time and event status for each patient were entered into Prism for Kaplan–Meier survival analysis. PFS and OS were analyzed separately, and differences between groups were evaluated using survival curve comparison analysis. Median survival times and *P* values were calculated where applicable.

Statistical significance was defined as *P* < 0.05, and nonsignificant results were annotated as “NS” on the figure.

## Results

### Evaluation of TEX TF-Ag-α, TEX^TF-Ag-α^ PD-L1, TEX LG3BP, and TEX^LG3BP^ PD-L1 for predicting response to anti–PD-1 immunotherapy in a discovery cohort of patients with NSCLC

In a discovery cohort of patients with NSCLC (*n* = 29), we compared TEX TF-Ag-α and TEX^TF-Ag-α^ PD-L1 with TEX LG3BP and TEX^LG3BP^ PD-L1 for predicting anti–PD-1 therapy response. LG3BP was selected as a comparator because it was reported as a promising biomarker for distinguishing benign pulmonary nodules from malignant lesions (30–33). Our previous findings showed that exosomal LG3BP discriminated lung cancer from normal controls with an AUC of 0.96 (29). The discovery cohort included 15 responders (CR or PR) and 14 nonresponders (SD or PD) according to RECIST criteria for objective response. The clinical characteristics of these patients are summarized in Table 1 and Supplementary Table S1 (No. 1–No. 29). Using Exo-PROS assay, we measured the levels of TEX TF-Ag-α, TEX^TF-Ag-α^ PD-L1, TEX LG3BP, and TEX^LG3BP^ PD-L1 at pretreatment baseline and at the end of the first treatment cycle (3–6 weeks after treatment). Our results demonstrated that TF-Ag-α enabled more effective TEX capture and improved predictive performance.

**Table 1.** Characteristics of patients with NSCLC receiving anti–PD-1 treatment.

*n* (%)				
Total patients	Discovery set (*n* = 29)		Validation set (*n* = 62)	
	Responder (*n* = 15)	Nonresponder (*n* = 14)	Responder (*n* = 25)	Nonresponder (*n* = 37)
Gender			​	
Female	8 (53)	7 (50)	14 (56)	23 (62)
Male	7 (47)	7 (50)	11 (44)	14 (38)
Age (years)			​	​
Mean	66.4	68.6	66.7	69.4
Median	66	68	65	71
Range	58–79	57–84	39–88	50–89
Stage			​	​
II	0 (0)	0 (0)	0 (0)	1 (3)
III	4 (27)	1 (7)	7 (28)	6 (16)
IV	11 (73)	13 (93)	18 (72)	30 (81)

**Figure 2.**
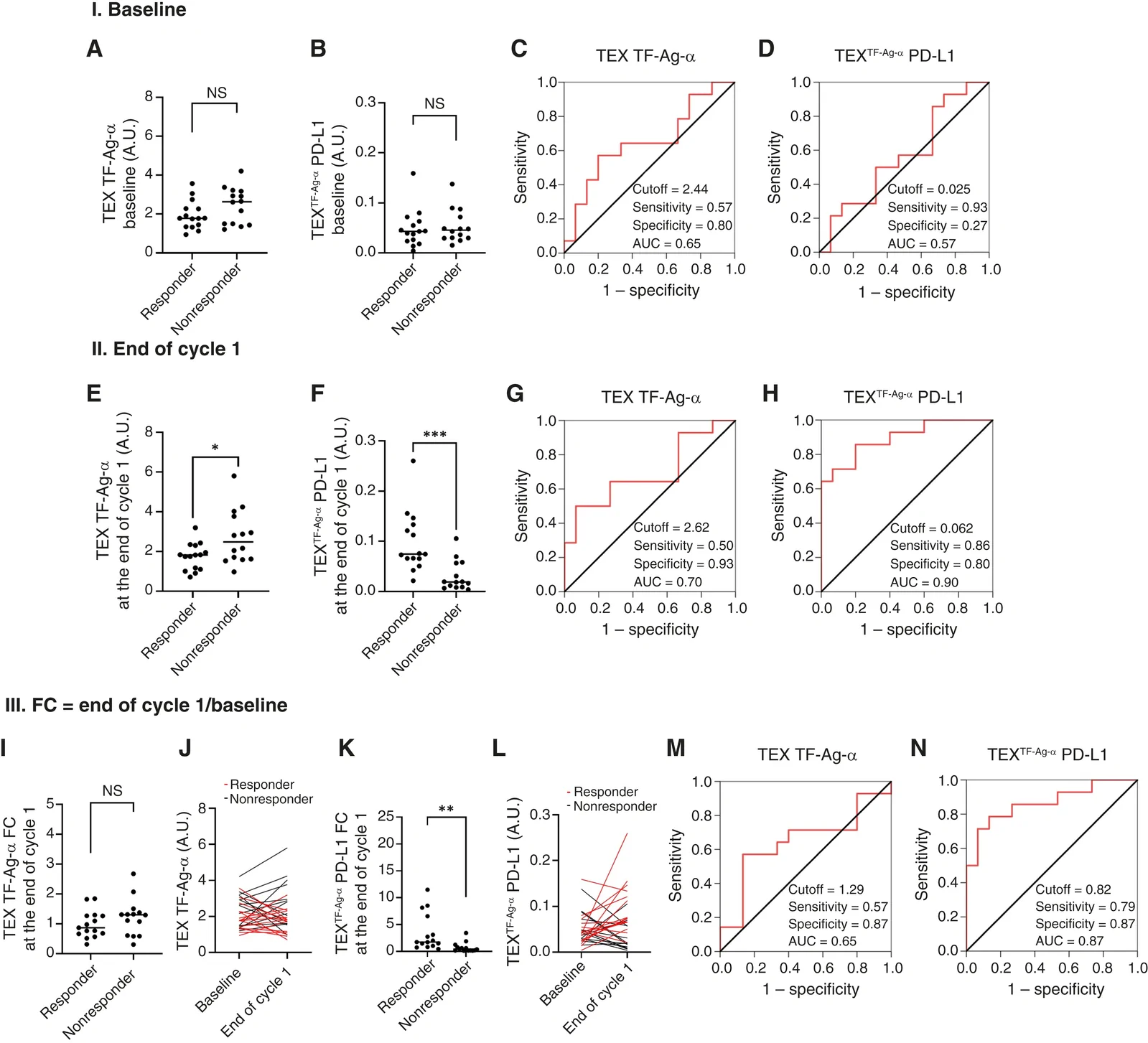
Evaluation of TEX TF-Ag-α and TEX^TF-Ag-α^ PD-L1 for predicting anti–PD-1 therapy response in a discovery cohort of patients with NSCLC (15 responders and 14 nonresponders). TEX TF-Ag-α and TEX^TF-Ag-α^ PD-L1 expression at pretreatment baseline (**A** and **B**), at the end of the first treatment cycle (**E** and **F**), and their FCs (**I–L**). ROC analysis of their predictive performance at baseline (**C** and **D**), at the end of the first treatment cycle (**G** and **H**), and FCs (**M** and **N**). *, *P* < 0.05; **, *P* < 0.01; ***, *P* < 0.001. NS, not statistically significant. A.U., arbitrary unit.

At pretreatment baseline, there were no significant differences in TEX TF-Ag-α or TEX^TF-Ag-α^ PD-L1 levels between responders (*n* = 15) and nonresponders (*n* = 14; Fig. 2A and B). ROC analysis showed that each biomarker alone had limited predictive performance. TEX TF-Ag-α distinguished responders from nonresponders with an AUC of 0.65, a sensitivity of 0.57, and a specificity of 0.80 at the cutoff of 2.44 (Fig. 2C). TEX^TF-Ag-α^ PD-L1 showed an AUC of 0.57, a sensitivity of 0.93, and a specificity of 0.27 at the cutoff of 0.025 (Fig. 2D). Combining the two biomarkers improved predictive performance, yielding an AUC of 0.77, a sensitivity of 0.64, and a specificity of 0.87 at the cutoff of 0.39 (Supplementary Fig. S1A).

At the end of the first treatment cycle, TEX TF-Ag-α levels were 0.64-fold lower in responders than nonresponders (1.74 vs. 2.72; *P* < 0.05), whereas TEX^TF-Ag-α^ PD-L1 levels were 3.13-fold higher (0.098 vs. 0.031; *P* < 0.001), effectively distinguishing the two groups (Fig. 2E and F). ROC analysis showed that each biomarker individually exhibited improved predictive performance relative to the pretreatment baseline. TEX TF-Ag-α distinguished responders from nonresponders with an AUC of 0.70, a sensitivity of 0.50, and a specificity of 0.93 at the cutoff of 2.62 (Fig. 2G). TEX^TF-Ag-α^ PD-L1 demonstrated an AUC of 0.90, a sensitivity of 0.86, and a specificity of 0.80 at the cutoff of 0.062 (Fig. 2H). Combining the two biomarkers yielded predictive performance comparable with TEX^TF-Ag-α^ PD-L1 alone, with an AUC of 0.88, a sensitivity of 0.86, and a specificity of 0.80 at the cutoff of 0.59 (Supplementary Fig. S1B).

Dynamic changes in biomarker levels were assessed by calculating FCs between the end of the first treatment cycle and the pretreatment baseline. No significant difference in TEX TF-Ag-α FC was observed between responders and nonresponders; the mean TEX TF-Ag-α FCs were 0.98 in responders and 1.23 in nonresponders (Fig. 2I and J). In contrast, responders exhibited a significantly greater fold increase in TEX^TF-Ag-α^ PD-L1 levels compared with nonresponders (*P* < 0.01); the mean TEX^TF-Ag-α^ PD-L1 FCs were 3.49 in responders and 0.76 in nonresponders (Fig. 2K and L). ROC analysis showed that FC-based metrics for each biomarker demonstrated improved predictive performance compared with pretreatment baseline measurements and comparable predictive performance with measurements obtained at the end of the first treatment cycle. TEX TF-Ag-α FC distinguished responders from nonresponders with an AUC of 0.65, a sensitivity of 0.57, and a specificity of 0.87 at the cutoff of 1.29 (Fig. 2M). TEX^TF-Ag-α^ PD-L1 FC showed better predictive performance, with an AUC of 0.87, a sensitivity of 0.79, and a specificity of 0.87 at the cutoff of 0.82 (Fig. 2N). Combining the FCs of the two biomarkers yielded slightly improved predictive performance compared with TEX^TF-Ag-α^ PD-L1 FC alone, achieving an AUC of 0.88, a sensitivity of 0.86, and a specificity of 0.87 at the cutoff of 0.59 (Supplementary Fig. S1C).

**Figure 3.**
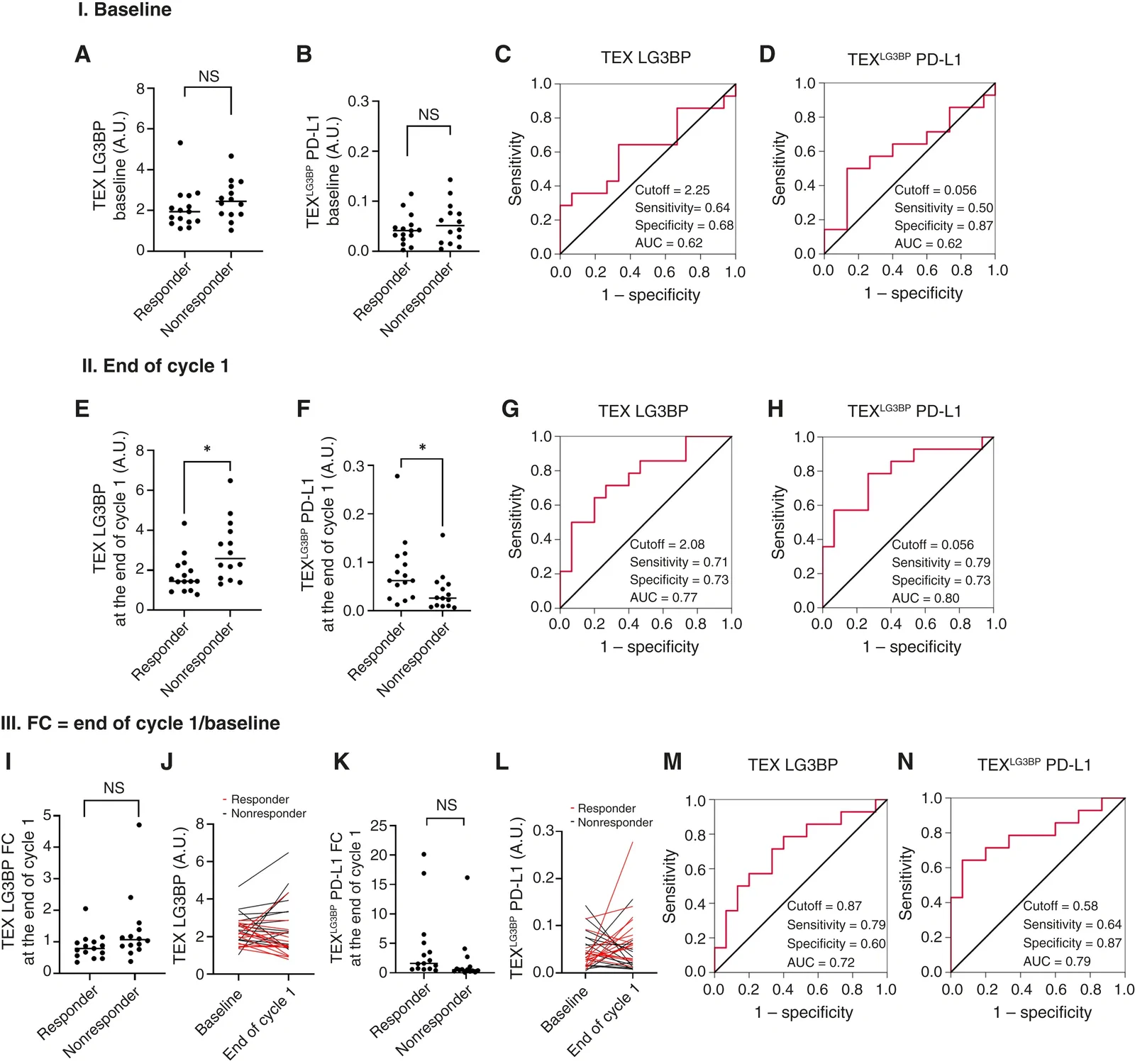
Evaluation of TEX LG3BP and TEX^LG3BP^ PD-L1 for predicting anti–PD-1 therapy response in a discovery cohort of patients with NSCLC (15 responders and 14 nonresponders). TEX LG3BP and TEX^LG3BP^ PD-L1 expression at pretreatment baseline (**A** and **B**), at the end of the first treatment cycle (**E** and **F**), and their FCs (**I–L**). ROC analysis of their predictive performance at baseline (**C** and **D**), at the end of the first treatment cycle (**G** and **H**), and FCs (**M** and **N**). *, *P *< 0.05. NS, not statistically significant. A.U., arbitrary unit.

In addition to TF-Ag-α, we used LG3BP to isolate TEX and measured TEX LG3BP and TEX^LG3BP^ PD-L1 levels in the same serum samples using the Exo-PROS assay (Fig. 3A–N; Supplementary Fig. S2). At baseline, no significant differences were observed in the TEX^LG3BP^ PD-L1 levels between responders and nonresponders (Fig. 3B). Baseline TEX^LG3BP^ PD-L1 exhibited slightly better predictive performance than baseline TEX^TF-Ag-α^ PD-L1 (AUC: 0.62 vs. 0.57; sensitivity: 0.50 vs. 0.93; specificity: 0.87 vs. 0.27; Fig. 3D). However, at the end of the first treatment cycle, even though significantly higher levels of TEX^LG3BP^ PD-L1 were observed in responders than nonresponders (0.082 vs. 0.037; *P* < 0.05; Fig. 3F), end-of-cycle 1 TEX^LG3BP^ PD-L1 showed inferior predictive performance compared with end-of-cycle 1 TEX^TF-Ag-α^ PD-L1 (AUC: 0.80 vs. 0.90; sensitivity: 0.79 vs. 0.86; specificity: 0.73 vs. 0.80; Fig. 3H). When dynamic FCs were assessed, no significant differences were observed in the TEX^LG3BP^ PD-L1 FCs between responders and nonresponders (Fig. 3K and L). The predictive performance of TEX^LG3BP^ PD-L1 FC was also lower than that of TEX^TF-Ag-α^ PD-L1 FC (AUC: 0.79 vs. 0.87; sensitivity: 0.64 vs. 0.79; specificity: 0.87 vs. 0.87; Fig. 3N). These results support TF-Ag-α as an effective biomarker for selective TEX capture and enhanced PD-L1 predictive performance.

The reason why TF-Ag-α is better than LG3BP is primarily because TF-Ag-α is a more cancer-specific biomarker than LG3BP. Although LG3BP contributes to tumor progression by promoting cell adhesion, migration, angiogenesis, EV signaling, and immune evasion, it is also broadly involved in immune regulation and inflammatory processes. As a result, LG3BP is less specific to cancer than TF-Ag-α. Consistent with this, TF-Ag-α exhibited higher selectivity for TEX and improved predictive accuracy of the TEX^TF-Ag-α^ PD-L1.

### Evaluation of TEX TF-Ag-α and TEX^TF-Ag-α^ PD-L1 for predicting response to anti–PD-1 immunotherapy in an internal validation cohort of patients with NSCLC

Encouraged by the findings from the discovery cohort, we next evaluated the predictive performance of TEX TF-Ag-α and TEX^TF-Ag-α^ PD-L1 in an internal validation cohort of patients with NSCLC (*n* = 62). The internal validation cohort comprised 25 responders (CR or PR) and 37 nonresponders (SD or PD). All patients were from the same institution, i.e., Roswell Park Comprehensive Cancer Center. The clinical characteristics of the patients are summarized in Table 1 and Supplementary Table S1 (No. 30–No. 91).

**Figure 4.**
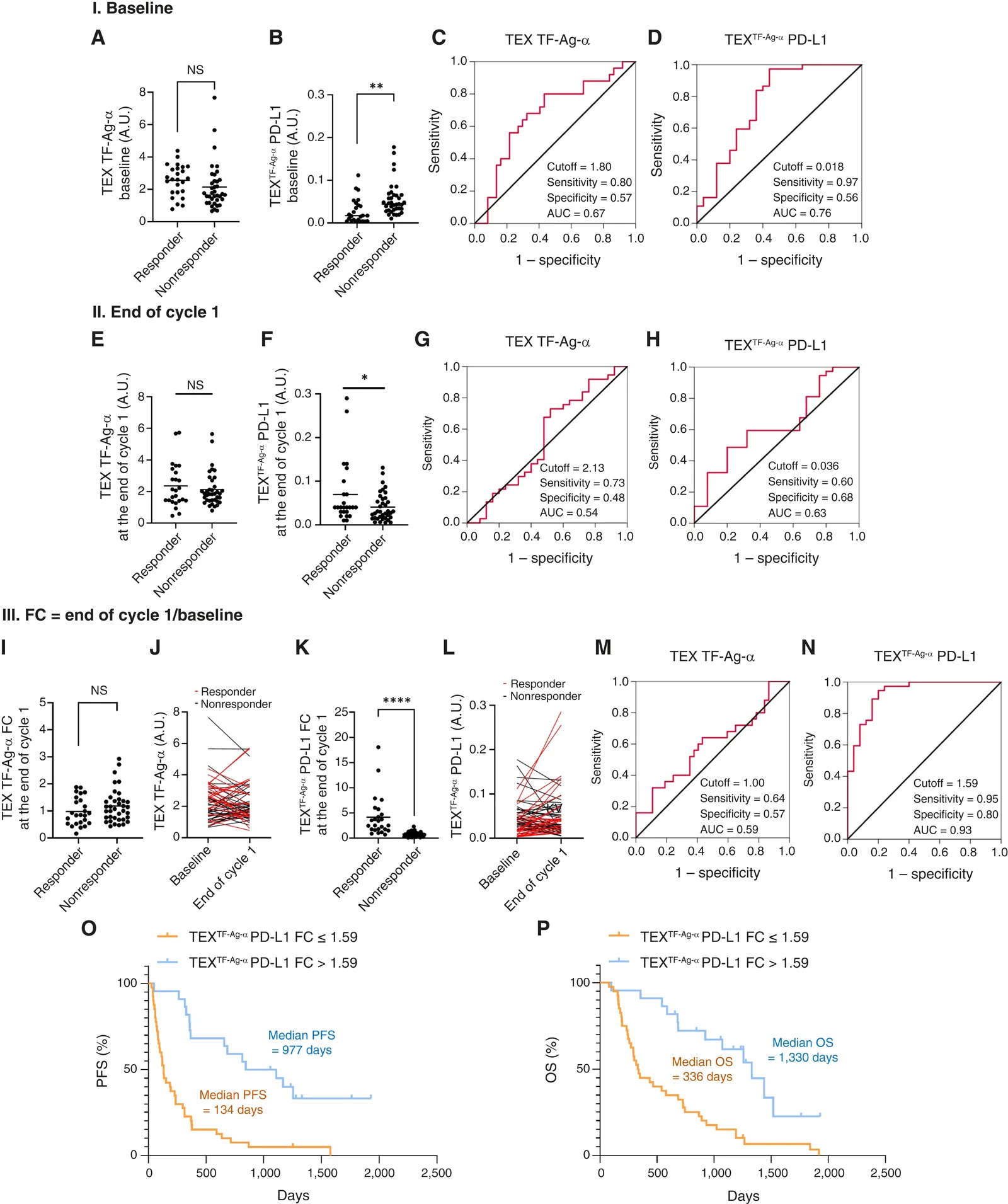
Evaluation of TEX TF-Ag-α and TEX^TF-Ag-α^ PD-L1 for predicting anti–PD-1 therapy response in a validation cohort of patients with NSCLC (25 responders and 37 nonresponders). TEX TF-Ag-α and TEX^TF-Ag-α^ PD-L1 expression at pretreatment baseline (**A** and **B**), at the end of the first treatment cycle (**E** and **F**), and their FCs (**I–L**). ROC analysis of their predictive performance at baseline (**C** and **D**), at the end of the first treatment cycle (**G** and **H**), and FCs (**M** and **N**). Kaplan–Meier survival analysis showed that patients with TEX^TF-Ag-α^ PD-L1 FC >1.59 had substantially longer PFS (**O**) and OS (**P**) compared with patients with TEX^TF-Ag-α^ PD-L1 FC ≤1.59 (*P* < 0.001). *, *P* < 0.05; **, *P* < 0.01; ****, *P* < 0.0001. NS, not statistically significant. A.U., arbitrary unit.

At the pretreatment baseline, TEX TF-Ag-α levels did not differ significantly between responders (*n* = 25) and nonresponders (*n* = 37; Fig. 4A), whereas TEX^TF-Ag-a^ PD-L1 levels were significantly lower in responders (*P* < 0.01; Fig. 4B). ROC analysis indicated that TEX TF-Ag-α distinguished responders from nonresponders with an AUC of 0.67, a sensitivity of 0.80, and a specificity of 0.57 at the cutoff of 1.80 (Fig. 4C). TEX^TF-Ag-α^ PD-L1 showed an AUC of 0.76, a sensitivity of 0.97, and a specificity of 0.56 at the cutoff of 0.018 (Fig. 4D). Combining the two biomarkers did not further improve predictive performance (Supplementary Fig. S3A); the AUC, sensitivity, and specificity remained the same as for TEX^TF-Ag-α^ PD-L1 alone.

At the end of the first treatment cycle, TEX TF-Ag-α levels were comparable between responders and nonresponders (Fig. 4E), whereas TEX^TF-Ag-α^ PD-L1 levels were 1.7-fold higher in responders (*P* < 0.05; Fig. 4F). ROC analysis showed that TEX TF-Ag-α distinguished responders from nonresponders with an AUC of 0.54, a sensitivity of 0.73, and a specificity of 0.48 at the cutoff of 2.13 (Fig. 4G) and TEX^TF-Ag-α^ PD-L1 demonstrated an AUC of 0.63, a sensitivity of 0.60, and a specificity of 0.68 at the cutoff of 0.036 (Fig. 4H). Combining the two biomarkers yielded a slightly better predictive performance, with an AUC of 0.67, a sensitivity of 0.92, and a specificity of 0.38 at the cutoff of 0.28 (Supplementary Fig. S3B). Compared with the discovery set, the predictive performance of TEX TF-Ag-α alone, TEX^TF-Ag-α^ PD-L1 alone, and their combination was all inferior, suggesting that biomarker levels measured at the end of the first treatment cycle may not serve as reliable direct predictive markers.

Dynamic changes in biomarker levels were assessed by calculating FCs between the end of the first treatment cycle and the pretreatment baseline. No significant difference in TEX TF-Ag-α FC was observed between responders and nonresponders; the mean TEX TF-Ag-α FCs were 0.98 in responders and 1.19 in nonresponders (Fig. 4I and J). In contrast, responders exhibited a significantly greater fold increase in TEX^TF-Ag-α^ PD-L1 levels compared with nonresponders (*P* < 0.0001); the mean TEX^TF-Ag-α^ PD-L1 FCs were 4.18 in responders and 0.82 in nonresponders (Fig. 4K and L). ROC analysis showed TEX TF-Ag-α FC distinguished responders from nonresponders with an AUC of 0.59, a sensitivity of 0.64, and a specificity of 0.57 at the cutoff of 1 (Fig. 4M), TEX^TF-Ag-α^ PD-L1 FC distinguished responders from nonresponders with an AUC of 0.93, a sensitivity of 0.95, and a specificity of 0.80 at the cutoff of 1.59 (Fig. 4N), and the combination yielded slightly improved predictive performance, achieving an AUC of 0.94, a sensitivity of 0.97, and a specificity of 0.84 at the cutoff of 0.44 (Supplementary Fig. S3C). Compared with pretreatment baseline measurements and those obtained at the end of the first treatment cycle, the TEX^TF-Ag-α^ PD-L1 FC showed significantly enhanced predictive performance. Moreover, relative to the observations in the discovery set, the TEX^TF-Ag-α^ PD-L1 FC also demonstrated improved predictive performance in the validation set. We note that the optimal cutoff for the TEX^TF-Ag-α^ PD-L1 FC was higher in the validation cohort than in the discovery cohort (1.59 vs. 0.82). This difference is likely due to the relatively small sample size of the discovery cohort, as cutoff values derived from small datasets are generally less stable. The higher cutoff identified in the validation cohort represents a more stringent criterion, requiring a greater increase in the TEX^TF-Ag-α^ PD-L1 FC to distinguish responders from nonresponders. Importantly, the cutoff values reported in this study represent the optimal cutoffs identified independently within each cohort and were not derived using a formal training-and-validation framework. A robust and clinically applicable cutoff should therefore be established and prospectively validated in larger, multicenter studies using a formal training and validation design.

TEX^TF-Ag-α^ PD-L1 FC was significantly associated with PFS and OS. Cox proportional hazards regression analysis demonstrated that, for PFS, the hazard ratio (HR) was 0.686 [95% confidence interval (CI), 0.557–0.844; *P* < 0.001], indicating that a higher TEX^TF-Ag-α^ PD-L1 FC was associated with a lower risk of progression and prolonged PFS. Specifically, each one-unit increase in FC corresponded to a 31.4% lower hazard of progression. For OS, Cox regression analysis yielded an HR of 0.698 (95% CI, 0.563–0.864; *P* < 0.001), indicating that each one-unit increase in FC was associated with a 30.2% lower hazard of death. Kaplan–Meier survival analysis further revealed that patients with a TEX^TF-Ag-α^ PD-L1 FC above the cutoff of 1.59 exhibited significantly longer PFS (Fig. 4O) and OS (Fig. 4P) than those with an FC below 1.59 (*P* < 0.001). The median PFS and OS in the high-FC group were 977 and 1,330 days, respectively, compared with only 134 and 336 days, respectively, in the low-FC group.

Collectively, these results indicate that the dynamic change in TEX^TF-Ag-α^ PD-L1 is a robust biomarker for predicting response to anti–PD-1 immunotherapy and patient outcomes (PFS and OS).

### Predictive performance comparison among TEX^TF-Ag-α^ PD-L1, total EV PD-L1, and tissue PD-L1

**Figure 5.**
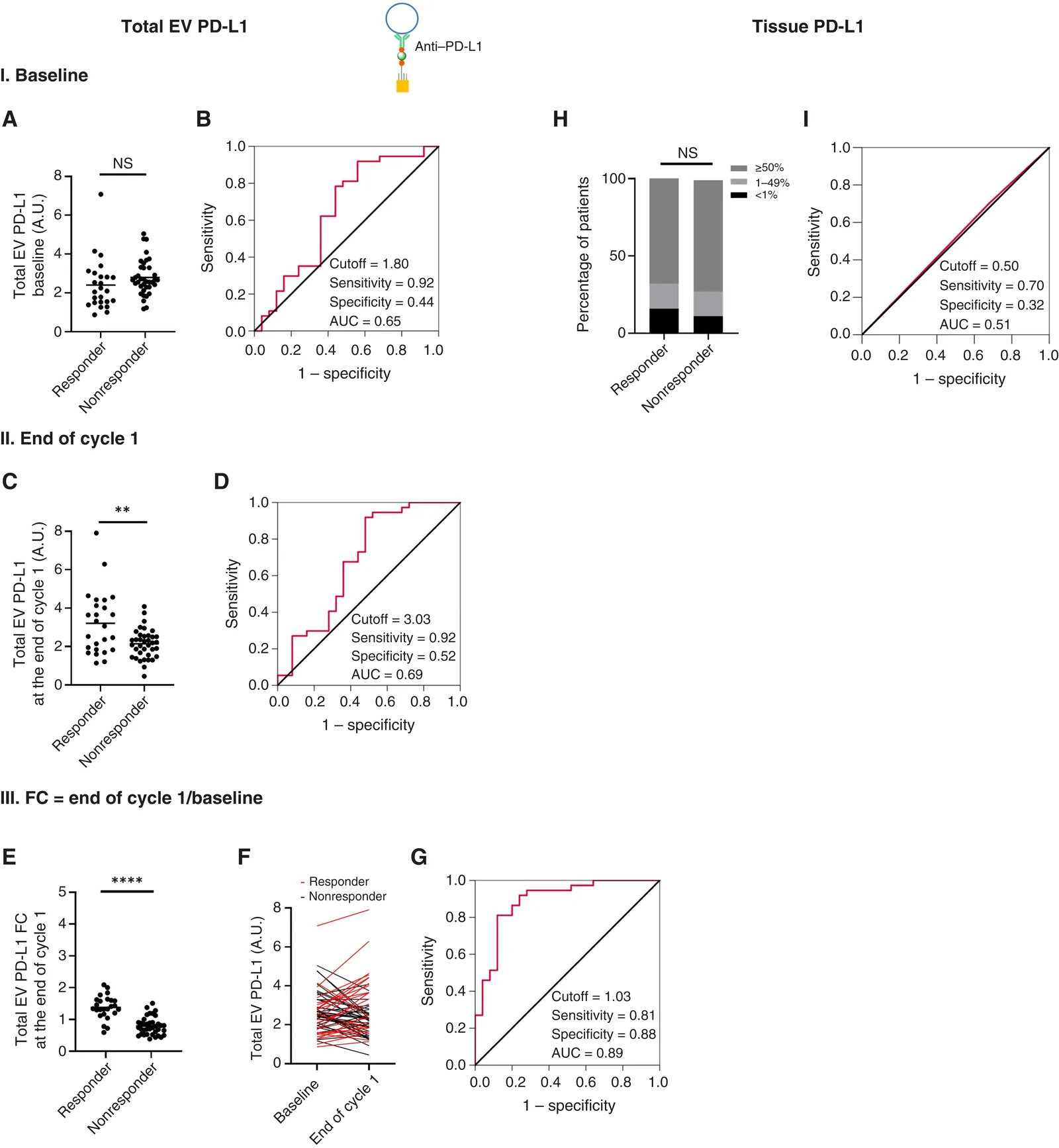
Predictive performance of total EV PD-L1 and tissue PD-L1 for response to anti–PD-1 immunotherapy (*n* = 25 responders and *n* = 37 nonresponders). Total EV PD-L1 expression at the pretreatment baseline (**A**), at the end of the first treatment cycle (**C**), and their FCs (**E** and **F**). Corresponding ROC analyses for distinguishing responders from nonresponders in **B**, **D**, and **G**, respectively. Tissue PD-L1 TPS (**H**) and corresponding ROC analysis (**I**) for distinguishing responders from nonresponders. **, *P* < 0.01; ****, *P* < 0.0001. NS, not statistically significant. A.U., arbitrary unit.

To determine whether TEX^TF-Ag-α^ PD-L1 demonstrates superior predictive performance for response to anti–PD-1 immunotherapy, we compared it with total EV PD-L1 and tissue PD-L1 (Fig. 5). Supplementary Figure S4 presents a side-by-side comparison of the predictive performance of TEX^TF-Ag-α^ PD-L1, total EV PD-L1, and tissue PD-L1. The analysis was conducted using the same patient cohort as the validation set, which included 25 responders and 37 nonresponders (Supplementary Table S1, No. 30–No. 91).

At the pretreatment baseline, TEX^TF-Ag-α^ PD-L1 levels were significantly lower in responders compared with nonresponders (*P* < 0.01; Fig. 4B), whereas total EV PD-L1 levels were comparable (Fig. 5A). At the end of the first treatment cycle, both biomarkers were significantly higher in responders and nonresponders (Figs. 4F and 5C). TEX^TF-Ag-α^ PD-L1 expression increased by 4.18-fold in responders but decreased by 0.82-fold in nonresponders (Fig. 4K and L). Similarly, total EV PD-L1 levels increased by 1.38-fold in responders whereas decreased by 0.8-fold in nonresponders (Fig. 5E and F).

Compared with total EV PD-L1, TEX^TF-Ag-α^ PD-L1 demonstrated superior discriminative performance, especially when dynamic FC was used as the biomarker. The ROC analysis showed that at the pretreatment baseline, TEX^TF-Ag-α^ PD-L1 distinguished responders from nonresponders with an AUC of 0.76, a sensitivity of 0.97, and a specificity of 0.56 (Fig. 4D), outperforming total EV PD-L1, which achieved an AUC of 0.65, a sensitivity of 0.92, and a specificity of 0.44 (Fig. 5B). At the end of the first treatment cycle, TEX^TF-Ag-α^ PD-L1 achieved an AUC of 0.63, a sensitivity of 0.60, and a specificity of 0.68 (Fig. 4H), slightly inferior to total EV PD-L1 (AUC: 0.69; sensitivity: 0.92, specificity: 0.52; Fig. 5D). When dynamic FC was used as the biomarker, TEX^TF-Ag-α^ PD-L1 FC yielded an AUC of 0.93, a sensitivity of 0.95, and a specificity of 0.80 (Fig. 4N), exceeding the performance of total EV PD-L1 FC (AUC: 0.89; sensitivity: 0.81; specificity: 0.88; Fig. 5G). The diagnostic performance of TEX^TF-Ag-α^ PD-L1 FC and total EV PD-L1 FC was compared using the DeLong test for AUCs, as well as comparisons of accuracy and Youden index (Supplementary Fig. S4O). No statistically significant differences were detected, potentially due to the limited sample size. Larger cohorts are needed for further validation. Notably, individual patient analysis (Figs. 4K vs. 5E in the main text; Supplementary Figs. S4E vs. S4L in the Supporting Information) revealed less overlap between responders and nonresponders for TEX^TF-Ag-α^ PD-L1 FC than total EV PD-L1 FC (highlighted by red rectangles in Supplementary Fig. S4E and S4L), suggesting improved discrimination at the individual-patient level. Collectively, these results demonstrate that selective capture of TEX enhances the predictive value of PD-L1 for anti–PD-1 therapy response.

Tissue PD-L1 TPSs were compared between responders and nonresponders (Fig. 5H). Among 25 responders, 17 had a PD-L1 TPS ≥ 50%, 4 had a TPS between 1% and 49%, and 4 had a TPS < 1%. Among 37 nonresponders, 26 had a PD-L1 TPS ≥ 50%, 8 had a TPS between 1% and 49%, and 3 had a TPS < 1%. The ROC analysis indicated that tissue PD-L1 TPS demonstrated limited predictive performance, with an AUC of 0.51, sensitivity of 0.70, and specificity of 0.32 at the cutoff of 0.50 (Fig. 5I), substantially lower than that of TEX^TF-Ag-α^ PD-L1 FC. This limitation was particularly apparent in patients with TPS < 1%, a clinically important subgroup that is traditionally more challenging to treat. Of the seven patients in this subgroup, four were responders and three were nonresponders. The finding that more than half of the patients with TPS < 1% achieved a clinical response highlights the limited utility of tissue PD-L1 as a predictive biomarker for immunotherapy response and underscores the need for more accurate and reliable biomarkers to guide patient selection. We acknowledge that tissue PD-L1 TPS is a static measurement obtained at a single time point, whereas TEX^TF-Ag-α^ PD-L1 FC reflects dynamic changes over time. Ideally, longitudinal assessment of tissue PD-L1 TPS would be performed to address the inherent limitation of comparing a static tissue biomarker with a dynamic FC biomarker. However, because tissue biopsy is an invasive procedure, longitudinal tissue specimens are frequently unavailable, making such analyses challenging in many patients.

## Discussion

Tissue PD-L1 TPS remains the most widely used biomarker to guide anti–PD-1 therapy in NSCLC. However, tissue PD-L1 showed limited predictive value (6, 7). In our cohort, tissue PD-L1 TPS showed an AUC of 0.51 in distinguishing responders from nonresponders, consistent with previously reported modest accuracy. This limitation likely reflects spatial heterogeneity, temporal evolution of PD-L1 expression, and the static nature of a single pretreatment biopsy.

In this study, we developed and validated a tumor-specific EV-based liquid biopsy assay to predict response to anti–PD-1 immunotherapy in patients with NSCLC. By leveraging the tumor-specific TF-Ag-α to selectively capture TEX, we demonstrated that dynamic changes in TEX^TF-Ag-α^ PD-L1 provide robust and reproducible predictive performance. Across independent discovery and validation cohorts, the FC in TEX^TF-Ag-α^ PD-L1 after the first treatment cycle consistently distinguished responders from nonresponders with high accuracy, achieving an AUC of 0.93, a sensitivity of 0.95, and a specificity of 0.80 at the cutoff of 1.59. When the FC in TEX^TF-Ag-α^ PD-L1 was combined with TEX TF-Ag-α FC, the predictive performance was further improved to an AUC of 0.94, sensitivity of 0.97, and specificity of 0.84. Importantly, this performance exceeded that of total EV PD-L1, underscoring the value of TF-Ag-α–positive, tumor-specific EV isolation for improving biomarker precision.

A major challenge in using EV PD-L1 as a biomarker is that PD-L1 is expressed not only by tumor cells but also by multiple immune cell populations. As a result, total EV PD-L1 measurements may reflect a mixture of tumor- and immune-derived vesicles, potentially confounding interpretation. Our results directly address this limitation. Compared with total EV PD-L1, TEX^TF-Ag-α^ PD-L1 consistently showed superior predictive performance at baseline and, more notably, when FC was used as the metric. These findings highlight the importance of selectively enriching TEX prior to biomarker quantification. TF-Ag-α, a tumor-specific glycoepitope exposed due to aberrant glycosylation in cancer cells, enables precise capture of TEX while minimizing background noise from nonmalignant sources. The comparison with LG3BP-based capture further supports this conclusion, as TF-Ag-α–based isolation outperformed LG3BP in predicting treatment response. Collectively, these results indicate that tumor specificity at the EV capture step is critical for maximizing the clinical utility of circulating PD-L1 measurements.

Another key finding of this study is that dynamic changes in TEX^TF-Ag-α^ PD-L1, rather than absolute levels measured at the pretreatment baseline or at the end of the first treatment cycle, provide the strongest predictive signal. In both the discovery and validation cohorts, the FC in TEX^TF-Ag-α^ PD-L1 consistently demonstrated high AUC, sensitivity, and specificity for predicting response to anti–PD-1 therapy. Interestingly, responders exhibited a significant increase (∼4.18-fold) in TEX^TF-Ag-α^ PD-L1 levels after initiation of anti–PD-1 therapy, whereas nonresponders showed a moderate decrease (∼20%). This observation agrees with results reported by other groups and may reflect an adaptive tumor response to immune activation (16, 18, 23). Effective PD-1 blockade can reinvigorate tumor-specific T cells, leading to enhanced immune pressure within the tumor microenvironment. In turn, tumor cells may upregulate PD-L1 expression and release PD-L1–bearing EVs as a compensatory immune evasion mechanism. Thus, an early increase in tumor-specific EV PD-L1 may serve as a pharmacodynamic marker of immune engagement and therapeutic activity. In contrast, the absence of such a dynamic response may indicate poor response or resistance to therapy. Additionally, TEX^TF-Ag-α^ PD-L1 FC was significantly associated with patient outcomes (PFS and OS) and effectively distinguished patients with longer PFS and OS from those with shorter PFS and OS, highlighting its potential as a prognostic biomarker. These findings align with the concept that early on-treatment biomarkers can outperform static pretreatment measurements in immunotherapy, particularly given the complex and evolving interactions between tumor and immune system.

Importantly, serum-based monitoring circumvents limitations of tissue biopsy and avoids the interpretive challenges of imaging, such as pseudoprogression and delayed radiographic responses. As a minimally invasive assay, this approach may be particularly valuable for patients with advanced or inoperable NSCLC in which repeated tissue biopsies are impractical. The Exo-PROS assay allows simultaneous quantification of TEX TF-Ag-α and TEX^TF-Ag-α^ PD-L1 from small serum volumes (as low as 10 μL). Our previous studies demonstrated that TEX can be directly captured from serum samples without requiring prior EV isolation or purification (27, 29). The Exo-PROS assay showed excellent reproducibility, with <10% interday, intraday, and interoperator variations (27, 29). In addition, the Exo-PROS assay features a user-friendly design, requiring only a micropipette to perform the assay. In the future, integration of the Exo-PROS biosensor with automated liquid handling systems may further streamline the assay workflow, improve throughout, and reduce costs, thereby supporting the feasibility for routine clinical implementation and benefiting large patient populations.

Several limitations should be acknowledged and addressed in future studies. First, although the study included independent discovery and validation cohorts, the overall sample size remains moderate. Findings such as the cutoff value for TEX^TF-Ag-α^ PD-L1 FC for determining response to anti–PD-1 immunotherapy should be considered suggestive rather than conclusive. Larger, multicenter prospective studies are needed to validate these findings and establish robust, clinically applicable cutoff values. Second, we only evaluated samples from the early stage of treatment. The utility of TEX^TF-Ag-α^ PD-L1 FC for monitoring long-term treatment response and irAEs remains to be established in later longitudinal samples. In patients without baseline samples, the TEX^TF-Ag-α^ PD-L1 FC between subsequent cycles, such as cycle 2 versus cycle 1, should be investigated as an alternative predictive measure. Third, TEX TF-Ag-α and TEX LG3BP showed limited predictive performance, potentially because samples were collected during early treatment stages, before tumor-associated changes were sufficiently reflected. Their utility for predicting and monitoring ICI responses should be evaluated using later longitudinal samples. Fourth, TF-Ag-α–based TEX capture may be less effective in tumors with low or absent TF-Ag-α expression. However, our previous studies detected TF-Ag-α in 84% of lung tumor tissues and in 93.3% of serum samples from patients with lung cancer (26, 27), suggesting that TF-Ag-α–negative tumors are uncommon and that most lung tumors and circulating TEX express sufficient TF-Ag-α to enable effective TEX capture. Moreover, because TEX^TF-Ag-α^ PD-L1 FC, rather than the TEX TF-Ag-α level, is the predictive biomarker, low TF-Ag-α expression is not expected to substantially affect its performance. Fifth, our study included only patients with NSCLC receiving PD-1 inhibitors; thus, the predictive value of TEX^TF-Ag-α^ PD-L1 with other ICIs, including PD-L1 inhibitors, remains to be established, as treatment differences may influence EV dynamics. Finally, future investigations should explore integrating TEX^TF-Ag-α^ PD-L1 dynamics with other circulating biomarkers, such as circulating tumor DNA or immune cell profiling, to develop multiparametric predictive models that may further improve predictive accuracy and clinical decision-making. This approach may help better distinguish patients with SD from those with PD, as these represent clinically distinct outcomes. In our validation set, patients with SD had significantly longer PFS and OS than those with PD (median PFS: 212 vs. 53 days; median OS: 707 vs. 179 days; *P* < 0.01). TEX^TF-Ag-α^ PD-L1 FC effectively distinguished responders from patients with SD (AUC = 0.94) and PD (AUC = 0.91) but could not distinguish SD from PD (AUC = 0.57; Supplementary Figs. S5–S7). The multiparametric predictive models may be useful in addressing this challenge.

### Conclusion

In summary, we demonstrated that selective capture of TEX using TF-Ag-α substantially enhanced the predictive value of EV PD-L1 for anti–PD-1 immunotherapy in NSCLC. Dynamic FCs in TEX^TF-Ag-α^ PD-L1 after the first treatment cycle provided a highly accurate, minimally invasive biomarker for early response assessment, outperforming both total EV PD-L1 and tissue PD-L1 TPS and correlating strongly with PFS and OS. Our results support using TEX^TF-Ag-α^ PD-L1 FC for early response stratification and outcome prediction, enabling earlier therapeutic adjustments and minimizing unnecessary exposure to irAEs. These findings warrant further validation in larger, prospective clinical trials.

## Supplementary Material

Supplementary Table S1 Table S1. Characteristics of nonsmall cell lung cancer patients receiving PD-1 checkpoint inhibitors.

Supplementary Figure S1 Figure S1. Evaluation of TEX TF-Ag-α and TEXTF-Ag-α PD-L1 combined biomarkers for predicting response to anti-PD-1 therapy in a discovery cohort of NSCLC patients.

Supplementary Figure S2 Figure S2. Evaluation of TEX LG3BP and TEXLG3BP PD-L1 combined biomarkers for predicting response to anti-PD-1 therapy in a discovery cohort of NSCLC patients.

Supplementary Figure S3 Figure S3. Evaluation of TEX TF-Ag-α and TEXTF-Ag-α PD-L1 combined biomarkers for predicting response to anti-PD-1 therapy in a validation cohort of NSCLC patients.

Supplementary Figure S4 Figure S4. Comparison of predictive performance of TEXTF-Ag-α PD-L1, total EV PD-L1 and tissue PD-L1 for response to anti-PD-1 immunotherapy in the validation cohort.

Supplementary Figure S5 Figure S5. ROC analysis of the predictive performance of TEX TF-Ag-α, TEXTF-Ag-α PD-L1, and their combination for differentiating responders from those with stable disease.

Supplementary Figure S6 Figure S6. ROC analysis of the predictive performance of TEX TF-Ag-α, TEXTF-Ag-α PD-L1, and their combination for differentiating responders from those with progressive disease.

Supplementary Figure S7 Figure S7. ROC analysis of the predictive performance of TEX TF-Ag-α, TEXTF-Ag-α PD-L1, and their combination for differentiating patients with stable disease from those with progressive disease.

## Data Availability

The data generated in this study are available upon reasonable request to the corresponding authors.
