# Supplementary material for: The Use of TF-Ag-α to Selectively Capture Tumor-Derived Extracellular Vesicles and Enhance the Predictive Performance of Extracellular Vesicle PD-L1 for Response to Immune Checkpoint Inhibitors
Source: Cancer Res Commun. 2026 Nov 2;6(11):2565–77. doi: 10.1158/2767-9764.CRC-26-0214 (PMC13725105; doi:10.1158/2767-9764.CRC-26-0214)
Supplement: Supplementary file 3 [file crc-26-0214_supplementary_figure_s3_suppsf3.pdf]

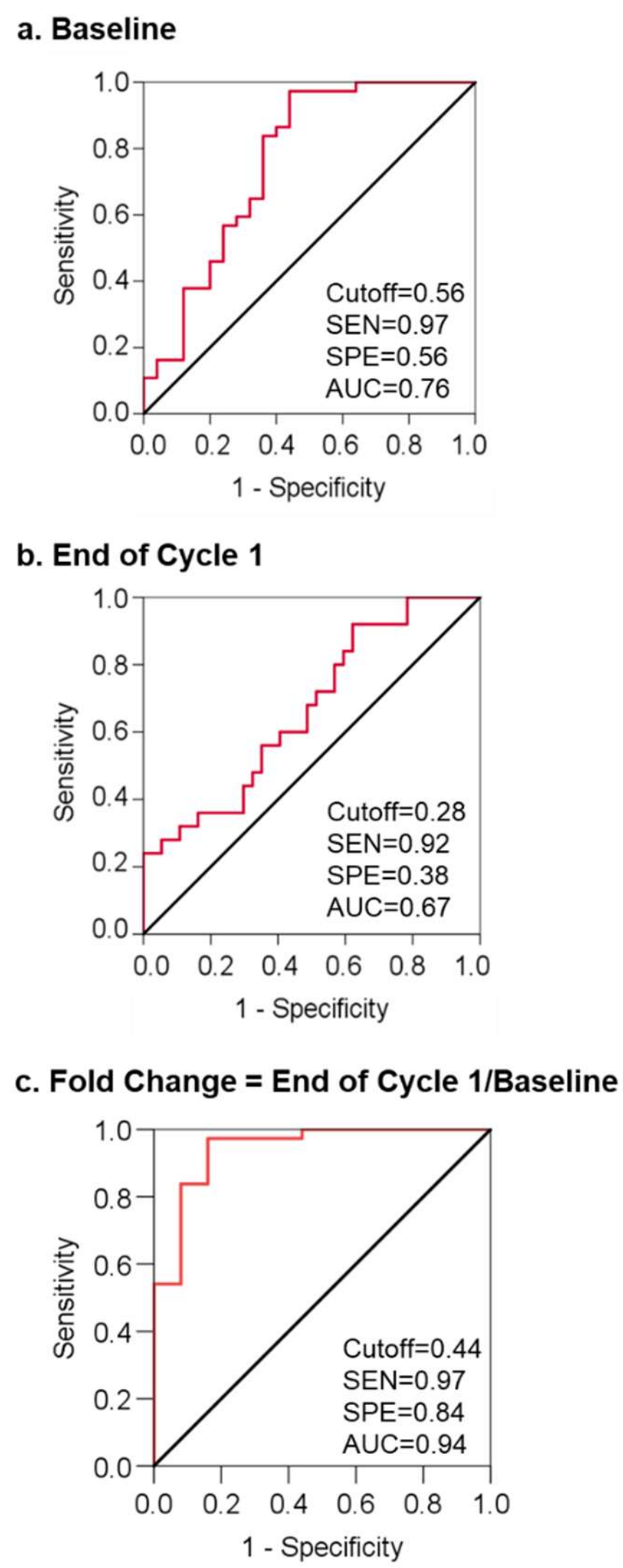

Supplementary Figure S3. Evaluation of TEX TF-Ag-α and $TEX^{TF\text{-}Ag\text{-}\alpha}$ PD-L1 combined biomarkers for predicting response to anti-PD-1 therapy in a validation cohort of NSCLC patients (n=25 responders; n=37 non-responders). ROC analysis of the predictive performance of the combination of (a) two baseline-based biomarkers (b) two end-of-cycle 1-based biomarkers and (c) two fold-change-based biomarkers for distinguishing responders from non-responders.
