# Supplementary material for: The Use of TF-Ag-α to Selectively Capture Tumor-Derived Extracellular Vesicles and Enhance the Predictive Performance of Extracellular Vesicle PD-L1 for Response to Immune Checkpoint Inhibitors
Source: Cancer Res Commun. 2026 Nov 2;6(11):2565–77. doi: 10.1158/2767-9764.CRC-26-0214 (PMC13725105; doi:10.1158/2767-9764.CRC-26-0214)
Supplement: Supplementary file 4 [file crc-26-0214_supplementary_figure_s4_suppsf4.pdf]

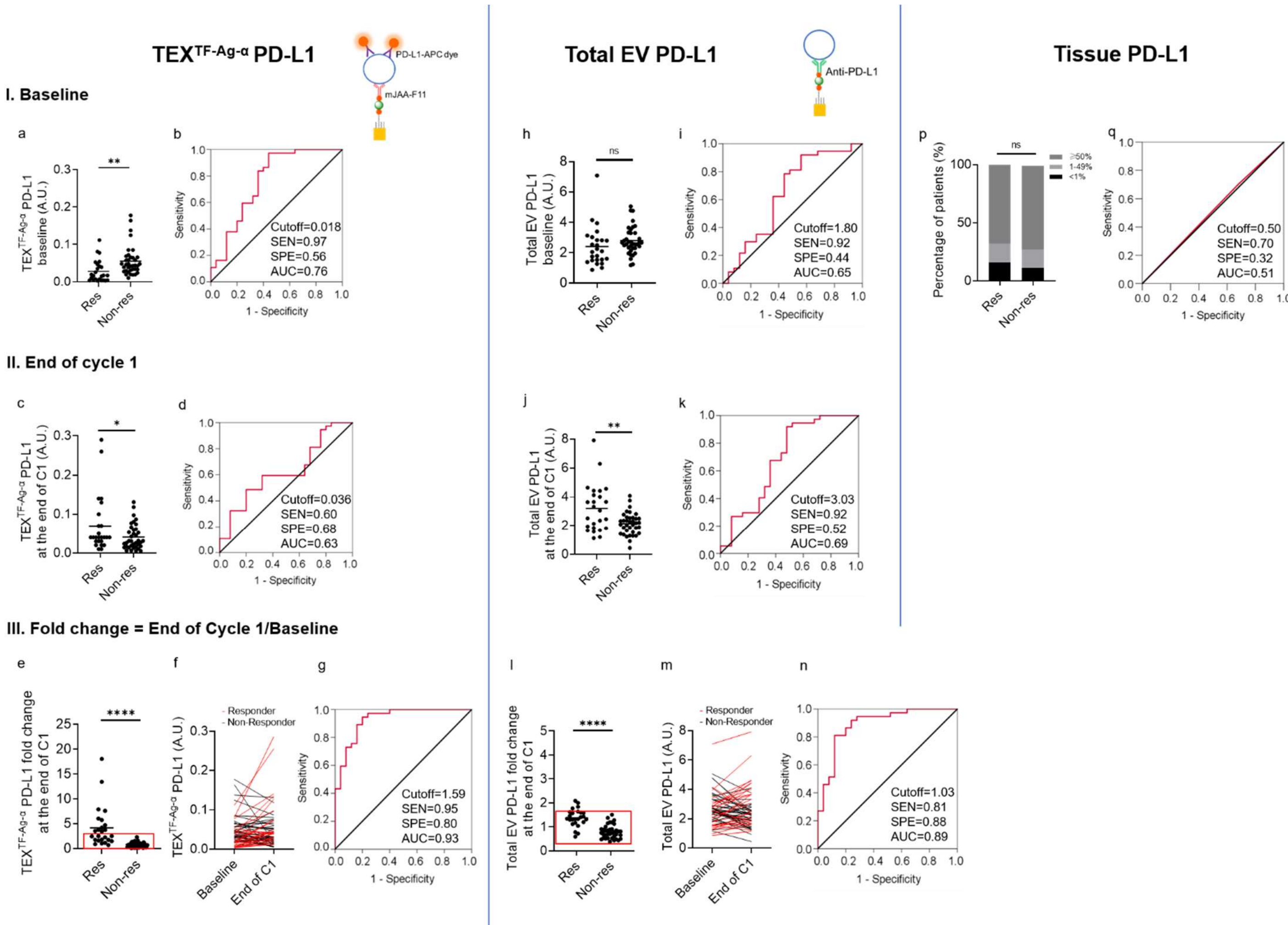

o

	AUC	Accuracy	Youden index
TEX$^{TF\text{-}Ag\text{-}\alpha}$ PD-L1 fold change	0.930	88.7%	0.746
Total EV PD-L1 fold change	0.890	83.9%	0.691
Significant difference	No, p=0.488	No, p=0.607	No, p=0.711
Method	DeLong's test	McNemar's test	Paired bootstrap

Supplementary Figure S4. Comparison of predictive performance of TEX$^{TF\text{-}Ag\text{-}\alpha}$ PD-L1, total EV PD-L1 and tissue PD-L1 for response to anti-PD-1 immunotherapy in the validation cohort (n = 25 responders; n = 37 non-responders). TEX$^{TF\text{-}Ag\text{-}\alpha}$ PD-L1 expression at the pre-treatment baseline (a), at the end of first treatment cycle (c) and as fold change (e, f). Corresponding ROC analyses for distinguishing responders from non-responders are shown in (b), (d) and (g), respectively. Total EV PD-L1 expression at the pre-treatment baseline (h), at the end of first treatment cycle (j) and as fold change (l, m). Corresponding ROC analyses for distinguishing responders from non-responders are shown in (i), (k) and (n), respectively. (o) No significant differences were observed between TEX$^{TF\text{-}Ag\text{-}\alpha}$ PD-L1 and total EV PD-L1 regarding their AUC, accuracy and Youden index. However, when individual patient data are examined, the overlap between responders and non-responders (highlighted by the red rectangles in e and l) is smaller when the TEX$^{TF\text{-}Ag\text{-}\alpha}$ PD-L1 fold change is used as the biomarker compared with total EV PD-L1 fold change. Tissue PD-L1 tumor proportion score (TPS, p) and corresponding ROC analysis (q) for distinguishing responders from non-responders.
