# Supplementary material for: The Use of TF-Ag-α to Selectively Capture Tumor-Derived Extracellular Vesicles and Enhance the Predictive Performance of Extracellular Vesicle PD-L1 for Response to Immune Checkpoint Inhibitors
Source: Cancer Res Commun. 2026 Nov 2;6(11):2565–77. doi: 10.1158/2767-9764.CRC-26-0214 (PMC13725105; doi:10.1158/2767-9764.CRC-26-0214)
Supplement: Supplementary file 5 [file crc-26-0214_supplementary_figure_s5_suppsf5.pdf]

I. Baseline

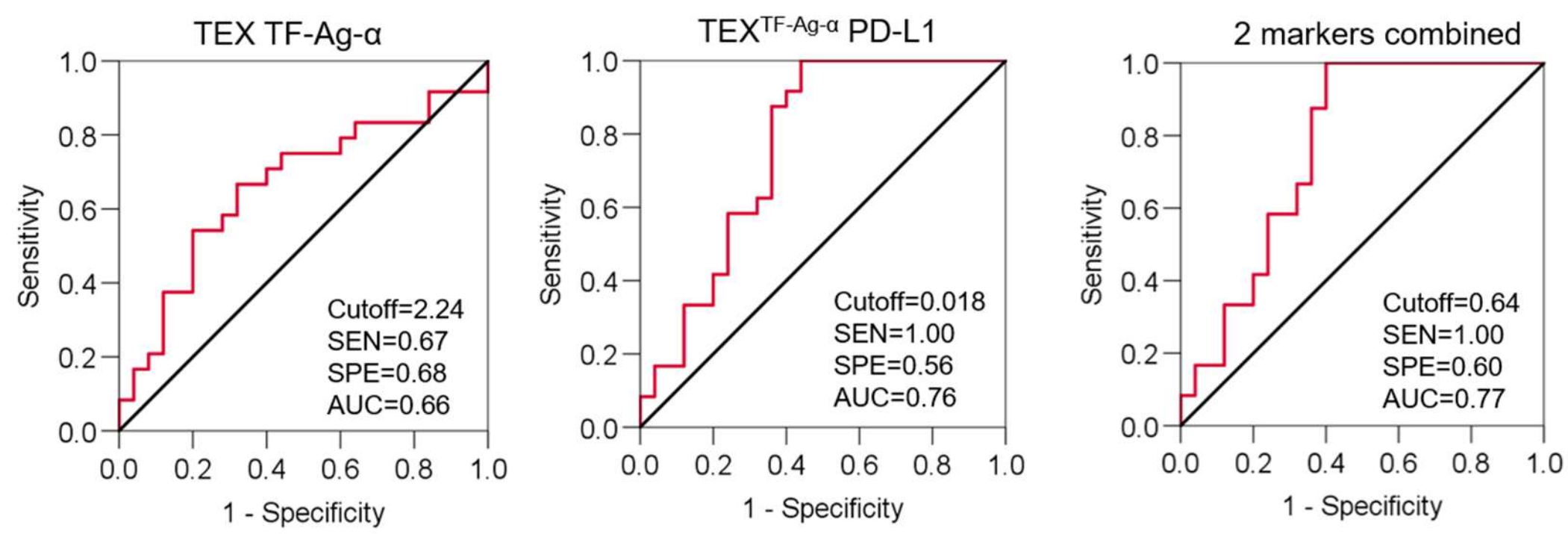

II. End of Cycle 1

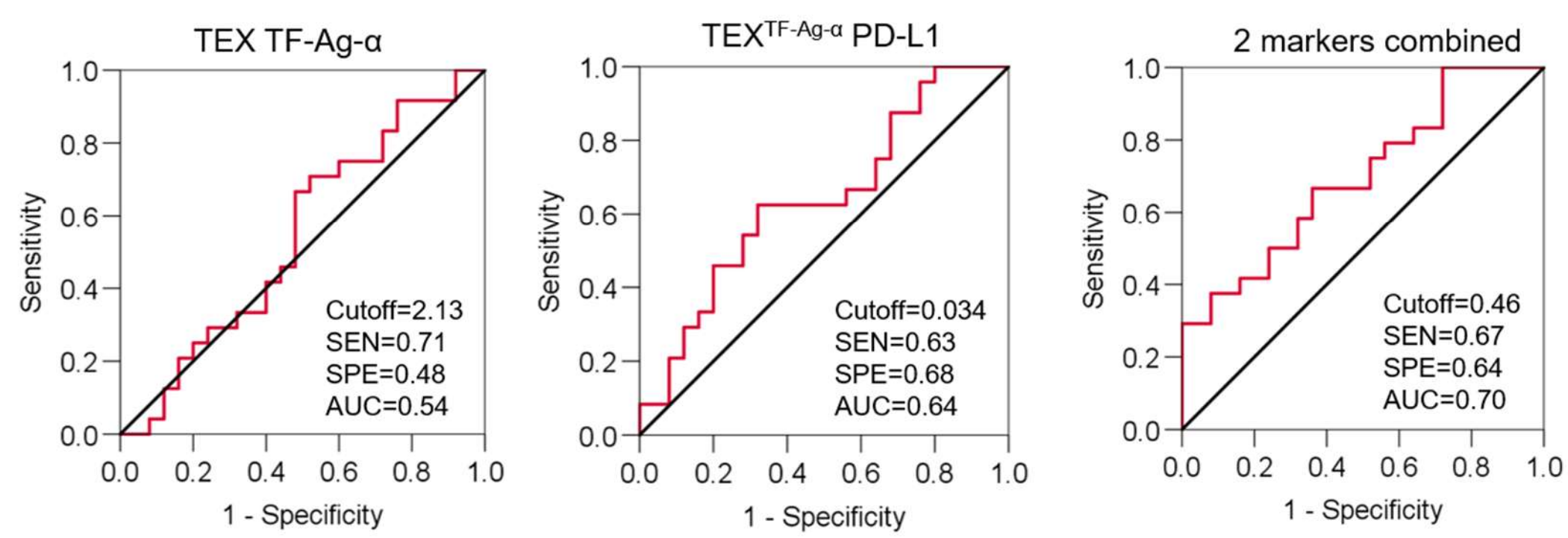

III. Fold Change = End of Cycle 1/Baseline

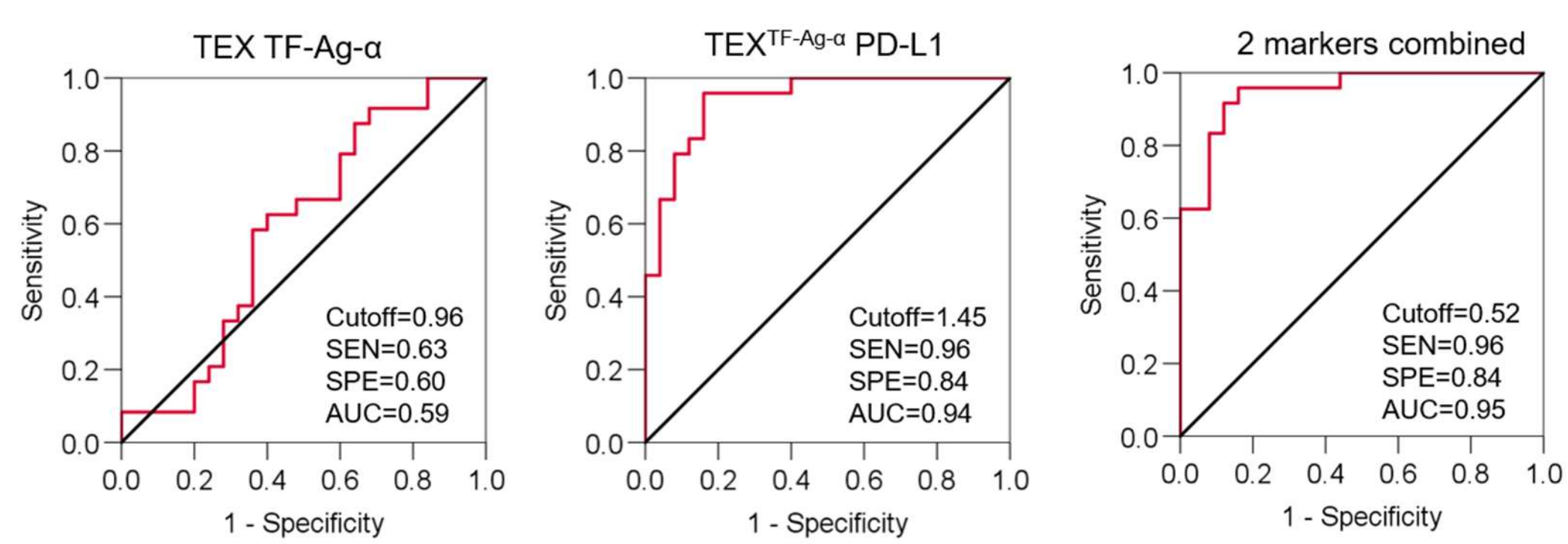

Supplementary Figure S5. ROC analysis of the predictive performance of TEX TF-Ag-α, $TEX^{TF-Ag-α}$ PD-L1, and their combination for differentiating responders (CR and PR; n=25) from those with stable disease (SD; n = 24). Marker expression at baseline, at the end of treatment cycle 1, and fold-change values were used to assess predictive performance.
