# Supplementary material for: The Use of TF-Ag-α to Selectively Capture Tumor-Derived Extracellular Vesicles and Enhance the Predictive Performance of Extracellular Vesicle PD-L1 for Response to Immune Checkpoint Inhibitors
Source: Cancer Res Commun. 2026 Nov 2;6(11):2565–77. doi: 10.1158/2767-9764.CRC-26-0214 (PMC13725105; doi:10.1158/2767-9764.CRC-26-0214)
Supplement: Supplementary file 7 [file crc-26-0214_supplementary_figure_s7_suppsf7.pdf]

I. Baseline

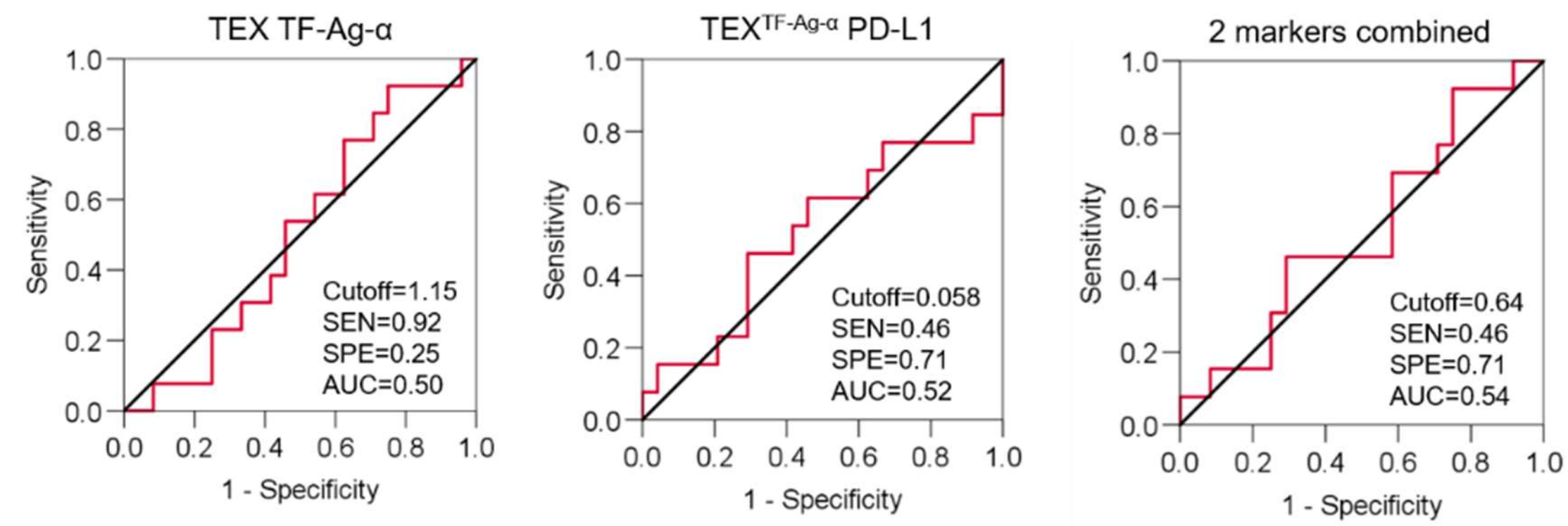

II. End of Cycle 1

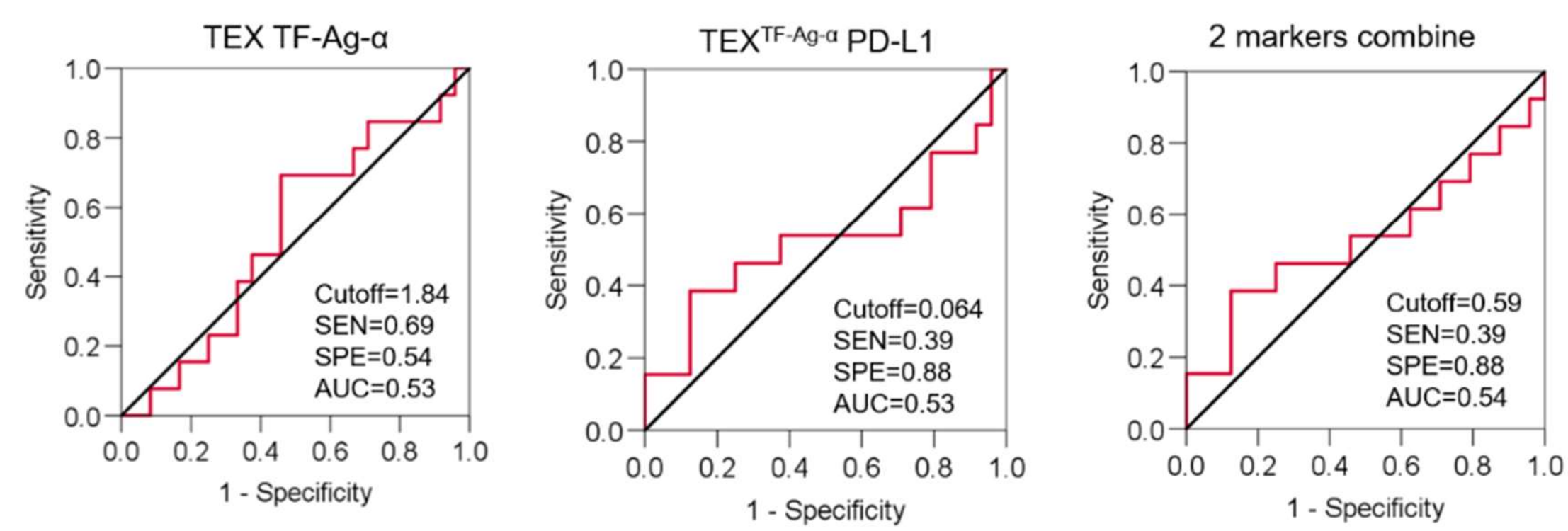

III. Fold Change = End of Cycle 1/Baseline

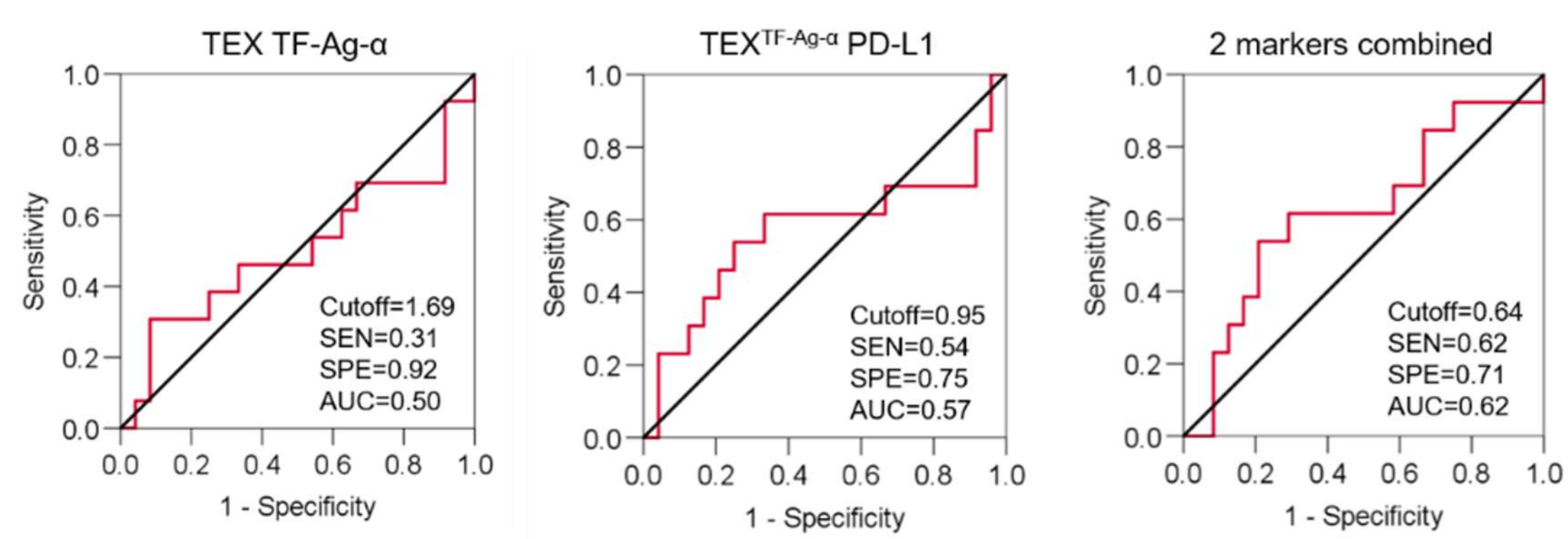

Supplementary Figure S7. ROC analysis of the predictive performance of TEX TF-Ag-α, TEX$^{TF-Ag-α}$ PD-L1, and their combination for differentiating patients with stable disease (SD; n = 24) from those with progressive disease (PD; n = 13). Marker expression at baseline, at the end of treatment cycle 1, and fold-change values were used to assess predictive performance.
