# Supplementary material for: The Use of TF-Ag-α to Selectively Capture Tumor-Derived Extracellular Vesicles and Enhance the Predictive Performance of Extracellular Vesicle PD-L1 for Response to Immune Checkpoint Inhibitors
Source: Cancer Res Commun. 2026 Nov 2;6(11):2565–77. doi: 10.1158/2767-9764.CRC-26-0214 (PMC13725105; doi:10.1158/2767-9764.CRC-26-0214)
Supplement: Supplementary file 8 [file crc-26-0214_supplementary_table_s1_suppst1.pdf]

Supplementary Table S1. Characteristics of nonsmall cell lung cancer patients receiving PD-1 checkpoint inhibitors. Discovery set: 1-29; Validation set: 30-91.

No.		Response*	Therapeutic regimen	Cancer subtype	Age Gender	Stage	Tissue PD-L1 TPS	PFS (day)	OS (day)	Prior local therapy
1	Discovery Set	Responder	Pembrolizumab Ramucirumab	Adenocarcinoma	66M	IV	0%	845	N/A	No
2		Responder	Pembrolizumab Carboplatin Paclitaxel	Squamous	66M	IV	>50%	910	N/A	No
3		Responder	Pembrolizumab Carboplatin Pemetrexed	Adenocarcinoma	72F	IV	1%	1190	N/A	No
4		Responder	Pembrolizumab Carboplatin Pemetrexed	Adenocarcinoma	62M	IV	20%	201	760	No
5		Responder	Pembrolizumab	Adenocarcinoma	77F	IV	90%	1234	N/A	No
6		Responder	Nivolumab Carboplatin Paclitaxel	Squamous	58M	IIIA	0%	1121	N/A	No
7		Responder	Pembrolizumab	Adenocarcinoma	79F	IV	>50%	658	N/A	No
8		Responder	Pembrolizumab Carboplatin Paclitaxel	Squamous	60F	III	0%	304	1284	No
9		Responder	Nivolumab Carboplatin Pemetrexed	Adenocarcinoma	59F	IIIA	1-49%	1095	N/A	No
10		Responder	Nivolumab Ipilimumab Carboplatin Pemetrexed	Adenocarcinoma	69M	IV	0%	183	N/A	No
11		Responder	Pembrolizumab Carboplatin Pemetrexed	Adenocarcinoma	66M	IV	1-49%	335	861	No
12		Responder	Cemiplimab	Adenocarcinoma	66F	IV	N/A	102	N/A	No
13		Responder	Nivolumab Cisplatin Pemetrexed	Adenocarcinoma	66F	IIIA	>50%	319	435	No
14		Responder	Pembrolizumab Carboplatin Paclitaxel	Squamous	64M	IV	>50%	936	N/A	No
15		Responder	Pembrolizumab Carboplatin Paclitaxel	Squamous	66F	IV	1-49%	199	344	No
16		Non-Responder	Pembrolizumab	Adenocarcinoma	84M	IV	10%	62	80	No
17		Non-Responder	Pembrolizumab	Adenocarcinoma	76F	IV	>50%	174	616	No
18		Non-Responder	Pembrolizumab Pemetrexed	Adenocarcinoma	72F	IV	1-49%	54	98	No
19		Non-Responder	Nivolumab	Adenocarcinoma	59F	IV	0%	27	600	No
20		Non-Responder	Nivolumab Ipilimumab	Squamous	75F	IV	0%	57	558	No
21		Non-Responder	Pembrolizumab	Adenocarcinoma	67F	IV	>50%	49	163	No
22		Non-Responder	Pembrolizumab Carboplatin Pemetrexed	Adenocarcinoma	67F	IV	0%	357	1045	No
23		Non-Responder	Pembrolizumab Carboplatin Pemetrexed	Adenocarcinoma	72F	IV	0%	271	507	No
24		Non-Responder	Pembrolizumab Carboplatin Paclitaxel	Squamous	57M	IV	1-49%	91	91	No
25		Non-Responder	Pembrolizumab Carboplatin Pemetrexed	Adenocarcinoma	69M	IV	0%	57	150	No
26		Non-Responder	Cisplatin Pemetrexed Pembrolizumab	Adenocarcinoma (colloid)	64M	IIIA	0%	1095	N/A	No
27		Non-Responder	Nivolumab Ipilimumab Carboplatin Paclitaxel	Squamous	60M	IV	0%	48	89	No
28		Non-Responder	Pembrolizumab Carboplatin	Adenocarcinoma	76M	IV	1-49%	120	N/A	No
29		Non-Responder	Pembrolizumab Carboplatin Pemetrexed	Adenocarcinoma	63M	IV	0%	171	403	No

30	Validation set	Responder	Pembrolizumab	Adenocarcinoma	57F	IV	70%	375	730	RT
31		Responder	Pembrolizumab	Adenocarcinoma	84M	IV	60%	314	339	No
32		Responder	Pembrolizumab	Adenocarcinoma	49F	IV	60%	1928	1928	No
33		Responder	Pembrolizumab	Adenocarcinoma	79F	IV	50%	133	161	RT
34		Responder	Pembrolizumab	Adenocarcinoma	75F	IV	90%	1762	1762	RT
35		Responder	Pembrolizumab	Squamous	61M	IV	>50%	360	588	RT
36		Responder	Pembrolizumab	Adenocarcinoma	88F	IIIA	50%	1109	1438	RT
37		Responder	Pembrolizumab	Adenocarcinoma	55M	IV	100%	329	542	RT
38		Responder	Pembrolizumab	Squamous	77F	IIIB	1-49%	265	356	RT
39		Responder	Pembrolizumab	Adenocarcinoma	52M	IV	>75%	1278	1278	RT
40		Responder	Pembrolizumab	Adenocarcinoma	66M	IV	100%	1331	1331	No
41		Responder	Pembrolizumab	Adenocarcinoma	63F	III	>50%	686	687	RT
42		Responder	Pembrolizumab	NSCLC with giant cell features	68M	IV	100%	1255	1330	No
43		Responder	Pembrolizumab	Squamous	64F	IIIA	80%	368	923	No
44		Responder	Pembrolizumab Carboplatin Pemetrexed	Adenocarcinoma	56F	IV	0%	1253	1253	RT
45		Responder	Pembrolizumab Carboplatin Pemetrexed	Adenocarcinoma	71F	IIIB	1-25%	814	1258	No
46		Responder	Pembrolizumab Carboplatin Pemetrexed	Adenocarcinoma	68M	IVB	20%	1255	1255	RT
47		Responder	Pembrolizumab Carboplatin Pemetrexed	Adenocarcinoma	65F	IIIA	1-25%	1169	1169	RT
48		Responder	Pembrolizumab Carboplatin Pemetrexed	Adenocarcinoma	61M	IV	>50%	314	351	No
49		Responder	Pembrolizumab Carboplatin Pemetrexed	Adenocarcinoma	39M	IV	100%	1057	1057	RT
50		Responder	Pembrolizumab Carboplatin Pemetrexed	Squamous	57F	IV	<1%	317	1071	No
51		Responder	Pembrolizumab Carboplatin Paclitaxel	Squamous	65M	IIIB	<1%	358	680	No
52		Responder	Pembrolizumab Ramucirumab	Adenocarcinoma	66M	IV	0%	845	N/A	No
53		Responder	Pembrolizumab	Adenocarcinoma	77F	IV	90%	1234	N/A	No
54		Responder	Pembrolizumab	Adenocarcinoma	79F	IV	>50%	658	N/A	No
55		Non-Responder	Pembrolizumab	Squamous	63F	IIIB	60%	195	1193	RT
56		Non-Responder	Pembrolizumab	Adenocarcinoma	89F	IV	50%	53	156	No
57		Non-Responder	Pembrolizumab Chemotherapy	Adenocarcinoma	59M	IV	0%	75	292	RT
58		Non-Responder	Pembrolizumab	Adenocarcinoma	74M	IV	70%	1579	1918	No
59		Non-Responder	Nivolumab	Adenocarcinoma	74F	IV	25%	229	466	RT
60		Non-Responder	Nivolumab	Squamous	74M	IV	2%	237	539	RT
61		Non-Responder	Pembrolizumab	Adenocarcinoma	73F	IV	50%	714	1841	No
62		Non-Responder	Pembrolizumab	Squamous	65M	IIIB	<50%	101	258	No
63		Non-Responder	Pembrolizumab	Adenocarcinoma	65F	IV	90%	89	247	RT
64		Non-Responder	Pembrolizumab	Squamous	68M	IIIB	50-75%	40	179	RT
65		Non-Responder	Pembrolizumab	Squamous	68F	IV	50-70%	96	168	RT
66		Non-Responder	Pembrolizumab	Squamous	60M	IV	90%	61	193	RT

67		Non-Responder	Pembrolizumab	Squamous	53F	IV	75-100%	46	271	No
68		Non-Responder	Pembrolizumab	Adenocarcinoma	59F	IV	>50%	237	687	No
69		Non-Responder	Pembrolizumab	Adenocarcinoma	76F	IV	50%	49	99	RT
70		Non-Responder	Pembrolizumab	Squamous	76F	IV	75%	115	867	RT
71		Non-Responder	Pembrolizumab	Squamous	72M	IV	10%	120	188	RT
72		Non-Responder	Pembrolizumab	Adenocarcinoma	51F	IV	50%	370	579	RT
73		Non-Responder	Pembrolizumab	Squamous	84M	IIB	>75%	593	1266	RT
74		Non-Responder	Pembrolizumab	Squamous	75F	III	>75%	39	163	RT
75		Non-Responder	Pembrolizumab	Adenocarcinoma	64M	IV	>50%	378	437	No
76		Non-Responder	Pembrolizumab	Adenocarcinoma	79F	III	>50%	1168	1519	RT
77		Non-Responder	Pembrolizumab	Adenocarcinoma	76F	IV	1-49%	61	80	RT
78		Non-Responder	Pembrolizumab	Squamous	70F	IV	100%	122	241	No
79		Non-Responder	Pembrolizumab	Adenocarcinoma	65F	IV	80%	29	119	RT
80		Non-Responder	Pembrolizumab	Adenocarcinoma	81M	IV	1-49%	84	1193	RT
81		Non-Responder	Pembrolizumab	Adenocarcinoma	59M	IV	>50%	154	294	RT
82		Non-Responder	Nivolumab	Squamous	57F	IV	>50%	637	745	RT
83		Non-Responder	Pembrolizumab	Adenocarcinoma	75M	IV	>50%	299	727	RT
84		Non-Responder	Pembrolizumab	Squamous	79F	IIIA	50%	134	300	No
85		Non-Responder	Pembrolizumab	Adenocarcinoma	71F	IV	100%	870	891	RT
86		Non-Responder	Pembrolizumab	Adenocarcinoma	77F	IV	>50%	188	333	RT
87		Non-Responder	Pembrolizumab	Poorly differentiated NSCLC, large cell carcinoma favored	50M	IV	<1%	42	195	No
88		Non-Responder	Pembrolizumab	Adenocarcinoma	66F	IV	<1%	65	234	No
89		Non-Responder	Pembrolizumab	Adenocarcinoma	78M	IV	70%	70	317	No
90		Non-Responder	Nivolumab	Adenocarcinoma	78F	IV	15%	83	1022	RT
91		Non-Responder	Pembrolizumab	Squamous	63F	IV	10%	135	932	No

*Response:
Responder: Complete response or partial response
Non-Responder: Stabel disease or progression

NSCLC: nonsmall cell lung cancer
RT: radiation therapy
